# Treating lung cancer with dynamic conformal arc therapy: a dosimetric study

**DOI:** 10.1186/s13014-017-0823-y

**Published:** 2017-06-02

**Authors:** Primož Peterlin, Karmen Stanič, Ignasi Méndez, Andrej Strojnik

**Affiliations:** Institute of Oncology Ljubljana, Zaloška 2, Ljubljana, SI-1000 Slovenia

**Keywords:** Dynamic conformal arc therapy, Film dosimetry, Lung cancer

## Abstract

**Background:**

Lung cancer patients are often in poor physical condition, and a shorter treatment time would reduce their discomfort. Dynamic conformal arc therapy (DCAT) offers a shorter treatment time than conventional 3D conformal radiotherapy (3D CRT) and is usually available even in departments without inverse planning possibilities. We examined its suitability as a treatment modality for lung cancer patients.

**Methods:**

On a cohort of 35 lung cancer patients, relevant dosimetric parameters were compared in respective DCAT and 3D CRT treatment plans. Radiochromic film dosimetry in an anthropomorphic phantom was used to compare both DCAT and 3D CRT dose distributions against their planned counterparts.

**Results:**

In comparison with their 3D CRT counterparts, DCAT plans equal or exceed the agreement between the calculated dose and the dose measured using film dosimetry. In dosimetric comparison, DCAT performed significantly better than 3D CRT in dose conformity to PTV and the number of monitor units used per plan, and significantly worse in dose homogeneity, mean lung dose and lung volume exposed to 5 Gy or more (V5Gy). No significant difference was found in the V20Gy value to lung, dose to 1 cm^3^ of spinal cord, and the mean dose to oesophagus. Improvements in V20Gy and V5Gy were found to be negatively correlated. DCAT plans differ from 3D CRT by exhibiting a moderate negative correlation between target volume sphericity and dose homogeneity.

**Conclusions:**

With respect to the agreement between the planned and the irradiated dose distribution, DCAT appears at least as reliable as 3D CRT. In specific conditions concerning the patient anatomy and treatment prescription, DCAT may yield more favourable dosimetric parameters. On average, however, conventional 3D CRT usually obtains better dosimetric parameters. We can thus only recommend DCAT as a complementary technique to the conventional 3D CRT.

**Electronic supplementary material:**

The online version of this article (doi:10.1186/s13014-017-0823-y) contains supplementary material, which is available to authorized users.

## Background

Lung cancer is the most commonly diagnosed cancer in males worldwide, accounting for 17% of the total new cancer cases and 24% of total cancer-related deaths [1]. In Slovenia, 1214 new lung cancer patients were diagnosed in 2012, 906 men and 308 women, which comprises 12% and 6% of all new cancer cases, respectively [2].

Radiotherapy is an important modality in the treatment of lung cancer either in curative or in palliative setting. In non-small cell lung cancer, curative radiotherapy includes patients with inoperable early stage and radiotherapy combined with concomitant chemotherapy for patients with locally advanced stage. Curative radiotherapy in combination with chemotherapy is also indicated for limited stage small cell lung cancer. Among new patients diagnosed with lung cancer in Slovenia in 2012, 43.5% received radiotherapy as part of their first line treatment [2].

Since its inception almost two decades ago, intensity-modulated radiation therapy (IMRT) has proved to be a great improvement to radiotherapy, allowing for better sparing of healthy organs and a higher dose to the tumour. However, as the individual treatment field segments do not encompass the whole treatment volume projection, a concern has arisen that the interplay between the movement of multileaf collimator (MLC) leaves and the organ movement (e.g., due to breathing motion) may lead to either overdosage or underdosage of the treated volume and/or the healthy tissue. The concern about the interplay between the MLC and organ motion applies to every form of radiotherapy in which only part of the treatment volume is irradiated at a time. This applies to all forms of intensity-modulated therapy, including advanced techniques such as volumetric modulated arc therapy (VMAT) and tomotherapy, the “field-in-field” technique, or “forward-planning IMRT” technique, as well as dynamic- or virtual wedges.

A statistical analysis of a simulated patient motion [3] has shown that due to organ motion, dose in any given voxel delivered in a single fraction deviates from its expected value, with its standard deviation reaching up to 10% of the expected dose value. By splitting the dose into several fractions, however, the probability density function of dose deviations quickly converges towards a Gaussian, and for a conventional fractionation (e.g., 30 fractions), the standard deviation for dose deviation drops down to 1% for a 5 mm organ motion amplitude. An experimental verification with a motor-driven phantom to simulate organ motion [4] was devised shortly afterwards. Using an ionization chamber, the authors confirmed their previous findings, and in addition found out that lowering the dose rate (i.e., 300 vs. 500 MU/min) reduces dose variation due to organ motion. Another experimental investigation which used film dosimetry has shown that underdosage as large as 6% may occur [5]. Early research in this area led to the recommendations on management of respiratory motion [6].

Recently, there has been a renewed interest in treating lung cancer with dynamic conformal arc therapy [7, 8]. Conformal arc therapy avoids the interplay effect altogether, since the field shape encompasses the complete projection of the treated volume from every direction. This, along with the shorter treatment time, is considered the advantage of DCAT over 3D CRT treatment plans employing “field-in-field” technique.

The purpose of the present study is twofold. First, we want to ascertain that the dose distribution in lungs delivered by dynamic conformal arc therapy (DCAT) exhibits at least as good agreement with the planned dose distribution as the conventional 3D conformal radiotherapy (3D CRT). Second, we want to examine and compare the relevant radiotherapy parameters in both DCAT and 3D CRT treatment plans.

## Methods

### Patient selection

Based exclusively on dosimetric criteria, 35 patients were included in the study. They were considered suitable for DCAT treatment if either the total dose was below the value considered to cause myelopathy of the spinal cord, or if the distance between the planned treated volume (PTV) and the spinal cord was estimated as sufficient so that the beams traversing the spinal cord would not add up to a dose contribution high enough to cause myelopathy.

### Target volume, critical organs, and dose prescription

**Fig. 1:**
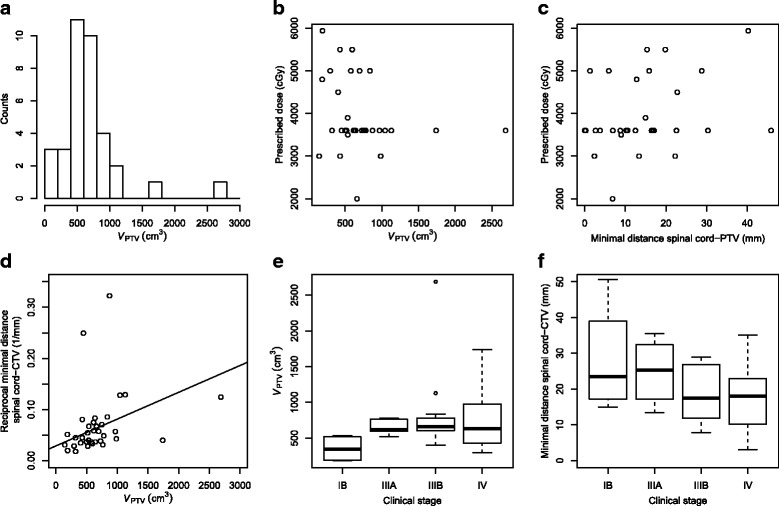
The metrics characterizing target size, its location with respect to the spinal cord, and its correlation with the clinical stage of the patients. Distribution of PTV by volume (**a**), the correlation between the PTV volume and the prescribed dose to PTV (**b**), the correlation between the minimal distance between PTV and the spinal cord and the prescribed dose to PTV (**c**), the correlation between the PTV volume and the reciprocal value of the distance between CTV and the spinal cord (**d**), and two boxplots showing the correlation between the clinical stage of the patient and their respective PTV volume (**e**) and the minimal distance between CTV and the spinal cord (**f**)

PTV was created by applying a 5–10 mm isotropic margin to clinical target volume (CTV), depending on the location of the tumour (apex vs. close to the diaphragm). Figure 1 shows the metrics characterizing the target size, its location and their relations with the prescribed dose and the clinical stage. PTV volumes range from 145.2 to 2686.5 cm^3^, with 70% of all cases corresponding to the 400– 1000 cm^3^ range (Fig. 1 a).

Lungs were contoured semi-automatically, based on Hounsfield number threshold, using the algorithm provided by the Eclipse v10.0 treatment planning system (Varian Medical Systems, Palo Alto, CA, USA). Both left and right lung were contoured as a single lung structure. The target was not excluded from the lungs; instead, there was an overlap between the lung structure and the target. On average, 68.9±45.5 cm^3^ (27.3±20.3*%*) of CTV and 214.4±85.3 cm^3^ (38.0±19.6*%*) of PTV overlapped with the lung structure.

The correlation between the PTV volume and the dose prescribed to PTV (Fig. 1 b) is not considered significant (Spearman’s rank correlation coefficient *ρ*=−0.229, *p*=0.185), although, as expected, the few cases of radical treatment doses included in the study were limited to PTV volumes below 1000 cm^3^. The standard palliative dose prescription for lung patients at our clinic is 36 Gy in 12 fractions; these cases comprise the bulk of the studied cases (Table 1).The correlation between the dose prescribed to PTV and the minimal distance between the PTV and the spinal cord (Fig. 1 c) is also not significant (*ρ*=0.254, *p*=0.140). On the other hand, significant correlation exists between the PTV volume and the reciprocal value of the minimal distance between the PTV and the spinal cord (*ρ*=0.525, *p*=0.001; Fig. 1 d). Boxplots show that the PTV volume generally increases with the clinical stage (Fig. 1 e), while the minimal distance between the CTV and the spinal cord decreases with the clinical stage (Fig. 1 f). PTV volume was computed using the treatment planning system (see below), while the minimal distance between either the PTV or the CTV and the spinal cord was computed using an in-house script written in Python which scanned the slices of DICOM RT Structure file for every patient.

**Table 1:** Total doses and fractionation schemes of the cases used in the study

TD (Gy)	Fractionation		#cases
36	12 ×	3 Gy	21
50	25 ×	2 Gy	3
30	10 ×	3 Gy	3
55	25 ×	2.2 Gy	2
59.4	27 ×	2.2 Gy	1
48	16 ×	3 Gy	1
45	18 ×	2.5 Gy	1
39	13 ×	3 Gy	1
35	14 ×	2.5 Gy	1
20	5 ×	4 Gy	1

### Treatment planning

Eclipse v10.0 was used as treatment planning system (TPS), using the AAA photon dose calculation algorithm. For the treatment, a 6 MV medical linac (Varian Unique Power; Varian Medical Systems) equipped with a Millenium 120-leaf multileaf collimator (MLC) was used.

Conventional 3D CRT treatment plans used a setup with 3 (25 out of 35 cases), 4 (9 cases) or 5 (1 case) principal fields. In 20 cases out of 35, one or more supplementary “field-in-field” fields (i.e., “forward-planning IMRT”) were used in addition to the principal treatment fields. Treatment fields were shaped by applying an 8 mm margin to the planning target volume (PTV) in the cranio-caudal direction and a 5 mm margin to the PTV in the transversal directions. Treatment planning restrictions used in our department generally follow QUANTEC [9]. For lungs the prescriptions are V20Gy<35*%*, V5Gy<60*%*, and MLD<15 Gy, for spinal cord, *D*_max_<50 Gy (prevails over target coverage), and for oesophagus, $\bar {D} < 34\;\text {Gy}$.

All DCAT plans were done identically, using a single arc with gantry running in a clockwise direction from 182° to 178°, with the control points evenly spaced every 4°, and the collimator tilted to 45°. An isotropic 5 mm margin was applied to PTV. In order to evaluate the dosimetric impact of the collimator angle, three additional settings for collimator angle were also tested: 0°, 30°, and 90°, yielding an overall number of 4×35 DCAT plans.

### Dose verification with film dosimetry

8 in×10 in Gafchromic EBT3 films (Ashland, Wayne, NJ, USA) from lots A03181301 and 04071601 were used for dose verification. The films were scanned before and 24±1 h after the irradiation using Epson Expression 10000XL flatbed scanner (Seiko Epson Corp., Nagano, Japan) in transmission mode, driven by Epson Scan v3.0 software. Images were acquired in 48-bit RGB mode using 72 dpi resolution with the image-processing options turned off. The doses were obtained using the Radiochromic.com (http://radiochromic.com/) web application [10], v1.1 through v2.5.

**Fig. 2:**
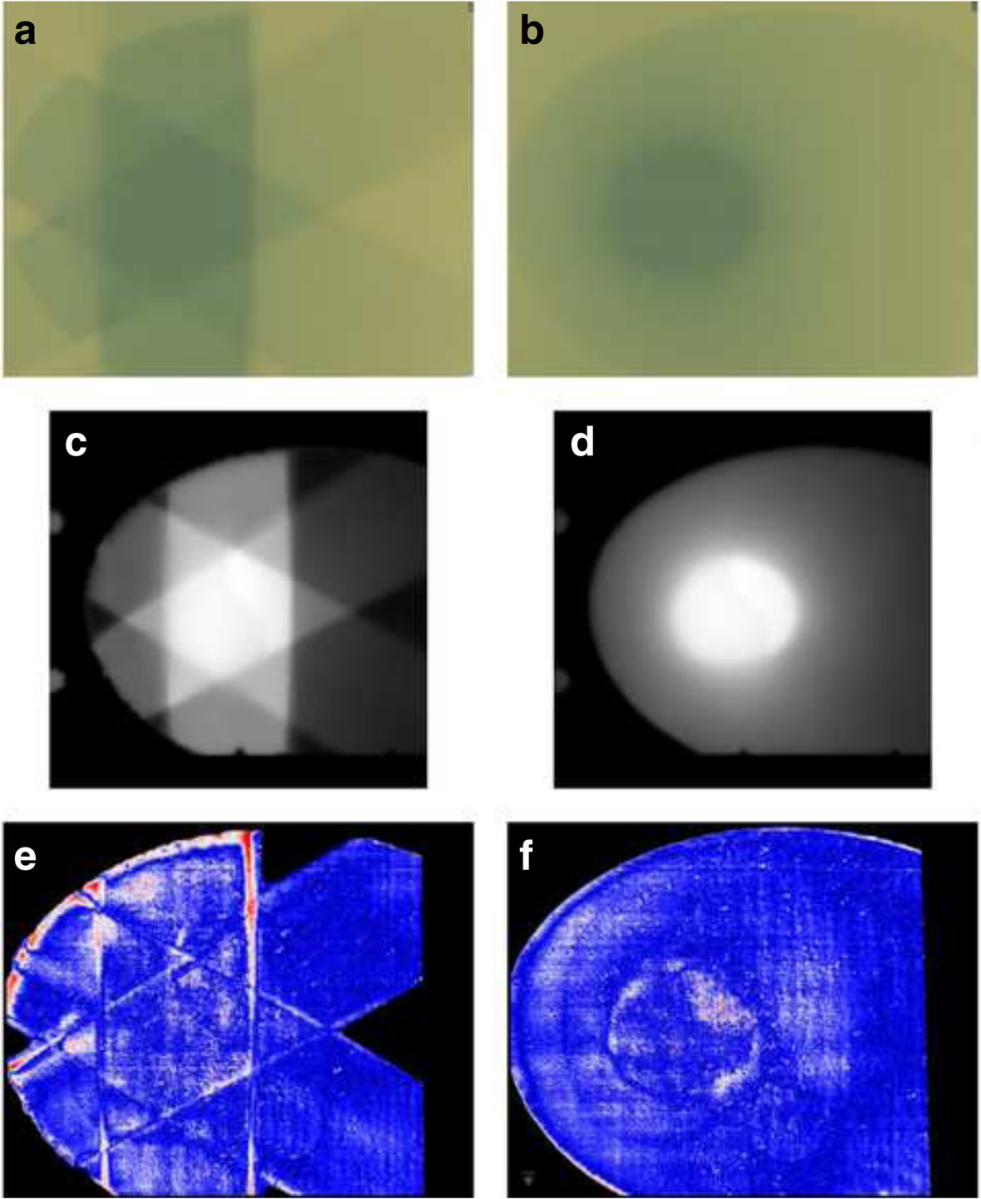
A comparison of the gamma index analysis for a 3D CRT (*left column*) and DCAT treatment plans (*right column*). *Top row*: irradiated radiochromic films (**a**, **b**), *middle row*: the corresponding dose planes exported from the treatment planning system (**c**, **d**), *bottom row*: gamma index maps (**e**, **f**). *Blue-coloured* regions correspond to areas with gamma index <1, *red-coloured* regions correspond to areas with gamma index >1

For dose verification purposes, 10 patients were randomly selected from the group. Both their 3D conformal plans and their DCAT plans were recalculated on the CIRS Thorax phantom (model 002LFC; Computerized Imaging Reference Systems, Norfolk, VA, USA), and the calculated dose plane taken 26 mm caudally from the isocenter coinciding with the phantom center. The chosen plane corresponding to the gap between plates marked #2 and #3 on the phantom was exported in DICOM RT Dose format. Again, a sheet of film was scanned first prior to being irradiated inside the CIRS Thorax phantom, and then again 24±1 h after the irradiation (Fig. 2, top row). The scanned images of the film before and after the irradiation, the exported dose plane (Fig. 2, middle row) and the daily output factor were imported into the Radiochromic.com web application to obtain the dose distribution and to conduct gamma index analysis.

### Dose calculation in different media

For each patient and for each treatment modality (3D CRT, DCAT), we delineated the regions with different electron densities (lung, soft tissue, bone) on the registered images of the calculated and the measured planar dose distributions. For every pixel, we calculated the relative dose difference (*D*_eval_−*D*_ref_)/*D*_ref_, where *D*_eval_ is the evaluation dose (calculated by the TPS) and *D*_ref_ is the reference dose (measured using the radiochromic film). Next we averaged the dose over the region for a given treatment plan, and finally we computed the systematic error *M*, its standard deviation *Σ*, and the random error *σ* [11].

### Data analysis

**Gamma index analysis.** Gamma index analysis [12] was used for comparing the reference and the evaluation dose distributions. For every point **r** referring to the reference dose distribution, one can define a function $ \gamma (\mathbf {r}) = \min _{\mathbf {r'}} \left (\sqrt { \delta ^{2}(\mathbf {r},\mathbf {r'})/\Delta D^{2} + r^{2}(\mathbf {r},\mathbf {r'})/\Delta d^{2} }\right)$, where *δ*(**r**,*r* ^′^)=*D* ^′^(*r* ^′^)−*D*(**r**) is the dose difference at point **r**, *r*(**r**,*r* ^′^)=|*r* ^′^−**r**| is the distance-to-agreement (DTA) at point **r**, *Δ* *D* and *Δ* *d* are the dose deviation and DTA criteria (commonly taken as 3% of the maximal dose and 3 mm), and **r** ^′^ is a point referring to the evaluation dose distribution. Common criteria for agreement between the two distributions are the ratio of points **r** for which *γ*(**r**)<1 holds, and the average value $\bar {\gamma }$.

**Dosimetric comparison of treatment plans.** Dose-volume histograms were exported from the treatment planning system and subsequently analysed with an in-house script written in GNU R (http://www.R-project.org/; [13]) using RadOnc package [14].

**Dose conformity.** A measure of dose conformity is the conformation number defined as CN=(*TV*_*RI*_/*TV*)·(*TV*_*RI*_/*V*_*RI*_), where *TV*_*RI*_ is the target volume covered by the reference isodose, *TV* is the target volume, and *V*_*RI*_ is the volume covered by the reference isodose [15]. In this case, the reference isodose was set to 95% of the prescription isodose. A higher CN value signifies a better conformity of the therapeutic dose to the target volume.

**Dose homogeneity.** A measure of dose homogeneity is the homogeneity index [16] defined as HI=(*D*_2_−*D*_98_)/*D*_nom_, where *D*_2_ is the dose to the 2% of the target volume (i.e., 2% of the target volume receives this dose or higher), *D*_98_ is the dose to the 98% of the target volume (i.e., 98% of the target volume receives this dose or higher), and *D*_nom_ is the nominal prescribed dose. A lower HI value indicates a more homogeneous target dose. Another measure of PTV homogeneity, dose homogeneity dispersion (DHD), is defined as the standard deviation of the absorbed dose covering PTV, divided by its mean value. A lower value of this parameter indicates a more homogeneous target dose. A similar parameter is the S-index proposed by Yoon [17].

**Target volume sphericity.** DCAT performs worse on highly concave target shapes with many protrusions. In order to quantify target volume shape, we have introduced the target volume sphericity parameter (Additional file 1). Sphericity *Ψ* is a dimensionless parameter which attains values close to 1 for near-spherical shapes, and falls towards 0 as the shape departs from the sphere.

### Target volume location

Our initial hypothesis was that DCAT works best with centrally located tumours, because treating peripheral targets would lead to hot spots in the areas proximal to the skin and cold spots in the distal areas, thus degrading dose homogeneity. In order to quantify tumour location, we introduced two geometrical parameters. The first one is the magnitude of the treatment field isocentre displacement from the patient origin (reference isocentre) in the transversal plane. Assuming that the patient origin is generally selected close to the centre of mass of an average cross-section, the displacement, calculated as a square root of the squares of displacements in the medio-lateral (*x*_ml_) and the antero-posterior direction (*x*_ap_), $R = \sqrt {x_{\text {ml}} + x_{\text {ap}}}$, is a measure of how centrally located is the target. The second parameter is the minimal distance between the CTV and the external contour (i.e., skin), which was computed by an in-house script written in Python which scanned the slices of DICOM RT Structure file for every patient.

## Results

### Dose verification with film dosimetry

Gamma index analysis [12] was used to compare the planar dose distributions for the 10 randomly selected pairs (conventional 3D CRT and DCAT) of treatment plans. The dose obtained from radiochromic film was taken as the reference distribution, and the corresponding dose plane exported from TPS was taken as the evaluation distribution. Several different values for the dose tolerance, positional tolerance, and dose threshold were used. Normalization to global *D*_max_ was used in all cases.

With the dose threshold set to 10*%* *D*_max_ and using 3% as dose tolerance and 3 mm as positional tolerance, the median value of the points with *γ*<1 was 94.8% (lower quartile 91.5%, upper quartile 97.88%) for conventional 3D CRT plans, and 97.3% (96.5%, 98.6%) for DCAT plans. The corresponding values of $\bar {\gamma }$ computed with the same parameters yielded a median of 0.38 (0.31, 0.42) for conventional 3D CRT plans and 0.26 (0.25, 0.30) for DCAT plans. Setting dose and positional tolerances to (2%, 2 mm), we obtain for *γ*<1 a median of 88.2% (85.1%, 91.3%) for conventional 3D CRT plans and 95.5% (94.5%, 98.6%) for DCAT plans. The corresponding values of $\bar {\gamma }(2\%,2\;\text {mm}$ are 0.49 (0.38, 0.55) for conventional 3D CRT and 0.33 (0.30, 0.38) for DCAT. Repeating the calculation with the dose threshold set to 60*%* *D*_max_ yielded results which are in agreement with the ones presented here, i.e., DCAT consistently resulted in slightly better results compared to conventional 3D CRT.

**Fig. 3:**
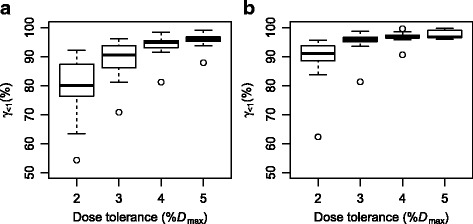
A comparison of the gamma index analysis for assessing the agreement between the measured and the calculated dose distribution. Using global *D*_max_, 10% dose threshold, 1 mm positional tolerance, and 2, 3, 4 and 5% dose tolerance, the ratio of points passing the *γ*<1 criterion was computed for conventional 3D CRT treatment plans (**a**) and DCAT treatment plans (**b**)

Figure 3 shows a comparison of the gamma index analysis for assessing the agreement between the measured and the calculated dose distribution. Using global *D*_max_, 10% dose threshold, 1 mm positional tolerance, and 2, 3, 4 and 5% dose tolerance, the ratio of points passing the *γ*<1 criterion was computed for conventional 3D CRT treatment plans (a) and DCAT treatment plans (b). We can see that overall, under the same conditions, DCAT treatment plans yield a higher ratio of points passing the *γ*<1 criterion. Both techniques reveal a single outlier (the same case with both conventional 3D CRT treatment plans and DCAT treatment plans) which exhibits a markedly worse results than the rest. With the exception of the said outlier, with DCAT, most plans pass the *γ*<1 criterion already with 1 mm positional and 3% dose tolerance.

The gamma value distribution maps (Fig. 2 e, f) show that both in conventional 3D CRT plans and in DCAT plans, the areas with a high gamma index are predominantly located along the phantom boundary where the treatment beams enter the phantom. These areas only reflect the inaccuracy of registration and/or the inacurracy of dose calculation in the buildup region. In conventional 3D CRT plans (Fig. 2 e), another area with high gamma index is along the lines which correspond to the boundaries of treatment fields, which can be attributed both to the error in phantom positioning as well as to a disagreement between the calculated and the measured dose in the penumbra region. In DCAT plans, dose gradients associated with the boundaries of treatment fields cannot be discerned. However, since most conventional 3D CRT treatments are fractionated, these dose gradients would be smeared out to some degree, which would likely diminish the difference in dose agreement between the two techniques even further.

### Dose calculation in different media

We analysed the dose difference distribution for the treatment plans which were previously selected for verification with the CIRS Thorax Phantom. The results for dose calculation in lung, bone and soft tissue are shown in Table 2. There are several results discernible from this table. First, the dose difference in the bone exhibits a systematic negative shift for both techniques (*M*=4.2*%* for conventional 3D CRT, 2.4% for DCAT), indicating that the treatment planning system underestimates the dose in bone regardless of the treatment modality and the individual patient geometry. Dose difference in the lung and the soft tissue does not exhibit such deviation, and their offsets are smaller than or comparable to *Σ*. Only in the lung does the Wilcoxon signed rank test show that we can convincingly (*p*<0.01) reject the null hypothesis that the difference between the pairs of mean dose follows a symmetric distribution centred at zero; both in bone (*p*=0.56) and in soft tissue (*p*=0.49), the results are inconclusive. Finally, lower values of random error *σ* for DCAT plans show that the 3D CRT plans generally produce a dose distribution with a larger standard deviation, which can be attributed to the regions with a high dose gradient. However, all the differences mentioned are unlikely to have a clinical significance.

**Table 2:** Systematic error *M*, its standard deviation *Σ*, and random error *σ* for the relative dose difference (*D*_eval_−*D*_ref_)/*D*_ref_, where *D*_eval_ is the evaluation dose (calculated by the TPS) and *D*_ref_ is the reference dose (measured using the radiochromic film), calculated for a group of 10 patients in three media with different electronic densities and separately for two different treatment planning techniques, conventional 3D CRT and DCAT

Medium	*M* (%)		*Σ* (%)		*σ* (%)	
	3D CRT	DCAT	3D CRT	DCAT	3D CRT	DCAT
Bone	-4.300	-2.400	2.177	1.154	4.749	1.218
Soft tissue	1.597	1.191	2.620	1.275	3.389	1.567
Lung	1.004	0.208	0.881	1.442	2.574	1.408

### Dosimetric comparison of treatment plans

**Fig. 4:**
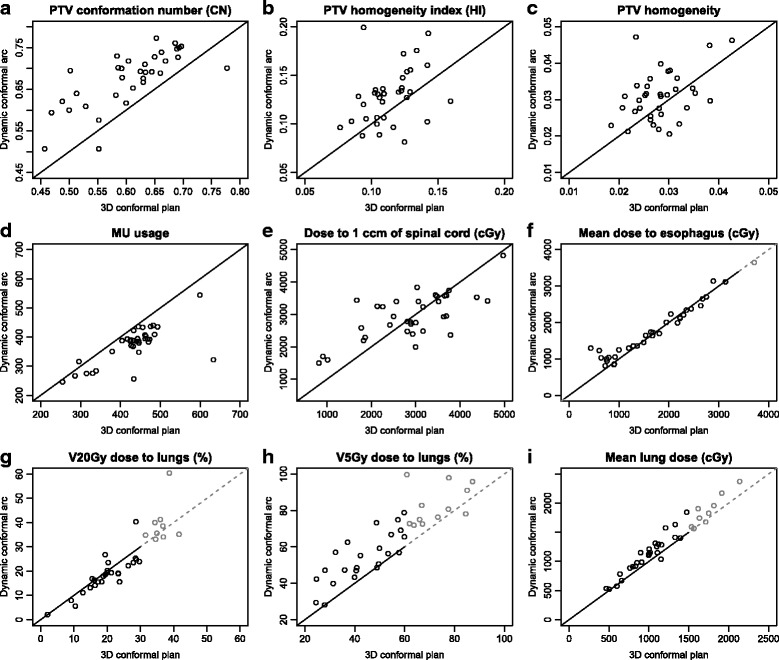
A comparison of several dosimetric parameters (PTV conformity (**a**), PTV homogeneity (**b**, **c**), MU usage (**d**), dose to the spinal cord (**e**), oesophagus (**f**) and lungs, **g**-**i**) in both types of treatment plan. An individual patient case is represented as a point in the diagram; its *x*-coordinate represents the parameter value in the conventional 3D CRT plan, and its *y*-coordinate represents the parameter value in the DCAT plan. The points shown in *gray* correspond to cases in which neither treatment plan meets the planning restrictions
- *Dose homogeneity (Fig.* 4 b). The Wilcoxon signed rank test shows a statistically significant difference of -0.016 (*p*=0.002, 95% CI [−0.023,−0.0071]) in favour of 3D CRT. Dose homogeneity dispersion (DHD) is shown in Fig. 4 c. The paired *t*-test shows a statistically significant difference of -0.0027 (*p*=0.024, 95% CI [−0.0051,−0.0004]) in favour of 3D CRT.
- *Monitor units (Fig.* 4 d). The Wilcoxon signed rank test shows a statistically significant difference of 49.9 MU (*p*<0.001, 95% CI [39.4,60.2]), indicating that as a rule, DCAT treatment plans use fewer monitor units than conventional 3D CRT treatment plans.
- *Spinal cord (Fig.* 4 e). The dose received by 1 cm3 of spinal cord is compared. The paired *t*-test shows a difference of -57.5 cGy in favour of conventional 3D CRT; the difference is however not statistically significant (*p*=0.630, 95% CI [−298,183]).
- *Oesophagus (Fig.* 4 f). The mean dose received by the oesophagus was compared. The Wilcoxon signed rank test shows a difference of -47.4 cGy in favour of conventional 3D CRT; however, the difference is not statistically significant (*p*=0.087, 95% CI [−113,8.8]). All treatment plans except one were clinically acceptable with respect to this parameter, with the mean dose to oesophagus below 34 Gy.
- *Lung.* Figure 4 g–i show a comparison of the three dosimetric parameters for lungs: percentage of lungs by volume exposed to the dose equal to 20 Gy or more (V20Gy), percentage of lungs by volume exposed to the dose 5 Gy or more (V5Gy), and the mean dose to lungs (MLD). For V20Gy, the Wilcoxon signed rank test shows a difference of 1.10% in favour of DCAT; however, the difference is not statistically significant (*p*=0.184, 95% CI [−0.58,2.33]). The paired *t*-test for V5Gy shows a statistically significant difference of -10.7% in favour of conventional 3D CRT (*p*<0.001, 95% CI [−14.1,−7.4]). Similarly, the paired *t*-test for MLD shows a statistically significant difference of -129 cGy in favour of conventional 3D CRT (*p*<0.001, 95% CI [−175,.83]). The cases in which neither treatment plan meets the planning restrictions are shown in gray. 86%, 66% and 74% of conventional 3D CRT treatment plans met the planning restrictions for V20Gy, V5Gy and MLD, compared to 80%, 46% and 66% of DCAT treatment plans. The cases in which the restrictions were not met required coordination between the radiation oncologist and the physicist/dosimetrist and subsequent plan modification.

Figure 4 shows the comparison of different dosimetric parameters in both types of treatment plan. An individual patient case is represented by a point in the diagram; its *x*-coordinate represents the parameter value in the conventional 3D CRT plan, and its *y*-coordinate represents the parameter value in the DCAT plan. In addition, the two-tailed paired Student’s *t*-test was performed in the cases in which the Shapiro-Wilk test showed normally distributed differences, and the non-parametric Wilcoxon matched pair signed rank sum test in the rest.

- *Dose conformity (Fig.* 4 a). The Wilcoxon signed rank test shows a statistically significant difference of -0.0726 in favour of DCAT (*p*<0.001, 95% CI [−0.0905,−0.055]). As the *TV*_*RI*_/*TV* ratio is approximately equal to 0.95 in both cases, the difference stems from a higher *TV*_*RI*_/*V*_*RI*_ ratio, or lower exposure of healthy tissue to a therapeutic dose, in the case of DCAT treatment plans.

### Correlations between dosimetric parameters for lungs

**Fig. 5:**
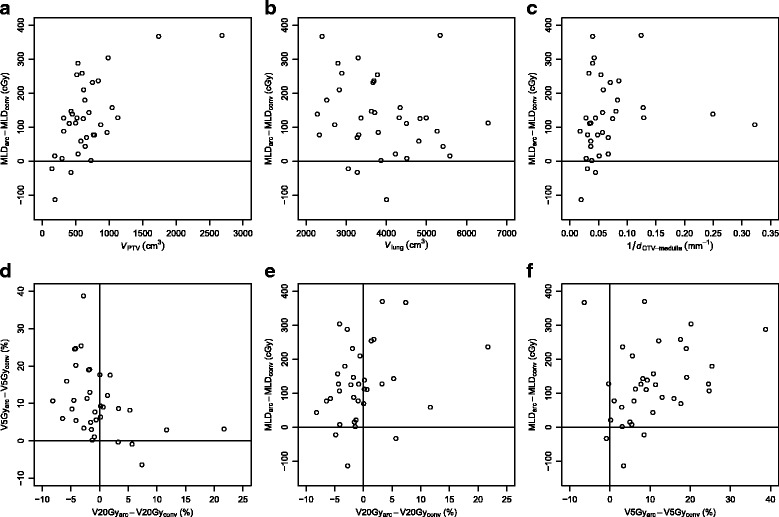
The dependence of the difference of the mean lung dose obtained by DCAT (MLD_*DCAT*_) and by the conventional 3D CRT treatment plan (MLD_3D*CRT*_) on the volume of the PTV (**a**), total lung volume (**b**), and the minimal distance between CTV and the spinal cord (**c**). The correlation between the differences in V5Gy and V20Gy (**d**), MLD and V20Gy (**e**), and MLD and V5Gy (**f**). An individual patient case is represented as a point in the diagram; its *x*- and *y*-coordinates represent its relevant parameter values

Figure 5 shows the correlation between dosimetric and geometric parameters as well as between different dosimetric parameters for lungs. Figure 5 a–c show the difference between the mean dose to lung (MLD) in DCAT treatment plans and MLD in conventional 3D CRT treatment plans with respect to the volume of PTV (*V*_PTV_), volume of lungs (*V*_lung_) and the reciprocal value of the minimal distance between CTV and the spinal cord (1/*d*_CTV−medulla_). Negative values of MLD_DCAT_−MLD_3D CRT_ signify an advantage of DCAT over conventional 3D CRT. Figure 5 a shows a moderate and significant (Spearman’s rank correlation coefficient *ρ*=0.472, *p*=0.005) correlation between MLD_DCAT_−MLD_3D CRT_ and *V*_PTV_, with DCAT being advantageous over conventional 3D CRT for small values of PTV volumes. Similarly, Fig. 5 c shows a weak yet significant (*ρ*=0.389, *p*=0.022) correlation between MLD_DCAT_−MLD_3D CRT_ and 1/*d*_CTV−medulla_, with DCAT being sometimes advantageous when the minimal distance between CTV and the spinal cord is large. No significant correlation was found between MLD_DCAT_−MLD_3D CRT_ and *V*_lung_ (Fig. 5 b). Likewise, no significant correlation was found between either V20Gy_DCAT_−V20Gy_3D CRT_ or V5Gy_DCAT_−V5Gy_3D CRT_ on the one hand and *V*_PTV_, *V*_lung_, and 1/*d*_CTV−medulla_ on the other (not shown).

Figure 5 d shows a moderate and significant (*ρ* = −0.489, *p*=0.003) negative correlation between V20Gy_DCAT_−V20Gy_3D CRT_ and V5Gy_DCAT_−V5Gy_3D CRT_. Again, negative values in Fig. 5 d–f signify an advantage of DCAT over conventional 3D CRT. Dosimetric improvements of DCAT for the V20Gy value for lung seems to be incompatible with the dosimetric improvement for the V5Gy value. No significant correlation was found between V20Gy_DCAT_−V20Gy_3D CRT_ and MLD_DCAT_−MLD_3D CRT_ (Fig. 5 e), while the correlation between V5Gy_DCAT_−V5Gy_3D CRT_ and MLD_DCAT_−MLD_3D CRT_ was found weak yet significant (*ρ*=0.398, *p*=0.018; Fig. 5 f). A dosimetric improvement in V5Gy value of one technique over the other is likely to be accompanied by an improvement of MLD.

### The choice of collimator angle

In this section, we examine the influence of the collimator angle on the observed dosimetric parameters. Table 3 shows a comparison for four different settings for collimator angle: 0°, 30°, 45°, and 90°. One can see that in general, the choice of collimator angle only minimally affects the value of dosimetric parameters. The largest difference between the largest and the smallest value (3.4%) occurs at V20Gy value for lung, followed by 2.5% D1cc difference for the spinal cord and 1.5% difference for mean lung dose. In all other observed parameters, the choice of collimator angle results in a difference less than 1%.

**Table 3:** A comparison of target dose homogeneity (HI, DHD), dose conformity (CN), D1cc for spinal cord, V20Gy and V5Gy values for lungs, and mean dose to lung and oesophagus obtained by dynamic conformal arc therapy plans with different values of collimator angle

		Dynamic conformal arc therapy (DCAT)			
Parameter	3D CRT	0°	30°	45°	90°
HI	0.144±0.018	0.132±0.027	0.133±0.027	0.133±0.027	0.134±0.028
DHD	0.028±0.005	0.032±0.007	0.032±0.007	0.032±0.007	0.032±0.007
CN	0.594±0.109	0.688±0.071	0.692±0.069	0.692±0.068	0.674±0.083
Spinal cord: D1cc (cGy)	2888±985	2916±723	2903±717	2896±711	2971±733
Lung: V20Gy (%)	23.9±9.4	22.9±12.1	23.1±12.2	23.3±12.3	23.7±12.5
Lung: V5Gy(%)	53.6±17.8	63.3±18.9	63.5±18.9	63.5±18.9	63.1±18.8
Lung: MLD (cGy)	1173±422	1270±466	1278±470	1284±473	1289±480
Oesophagus: D _*mean*_ (cGy)	1695±756	1750±756	1751±749	1752±748	1740±744

The corresponding values (mean ± sd) obtained by conventional 3D CRT are given for comparison

In an alternative analysis, we ranked the results obtained by different values of collimator angle at every patient. The results are similar to the ones above: the collimator set to 0° yielded the best result for HI in 20 cases out of 35 (20/35), DHD (18/35), V20Gy value for lungs (27/35), mean lung dose (28/35) and mean oesophagus dose (16/35). The collimator set to 45° yielded the best result for D1cc value for the spinal cord (23/35), and the collimator set to 90° yielded the best value for V5Gy value for lung (24/35). Even though some choices appear to be convincingly better, we need to reiterate that the differences are very small.

### Target volume sphericity

**Fig. 6:**
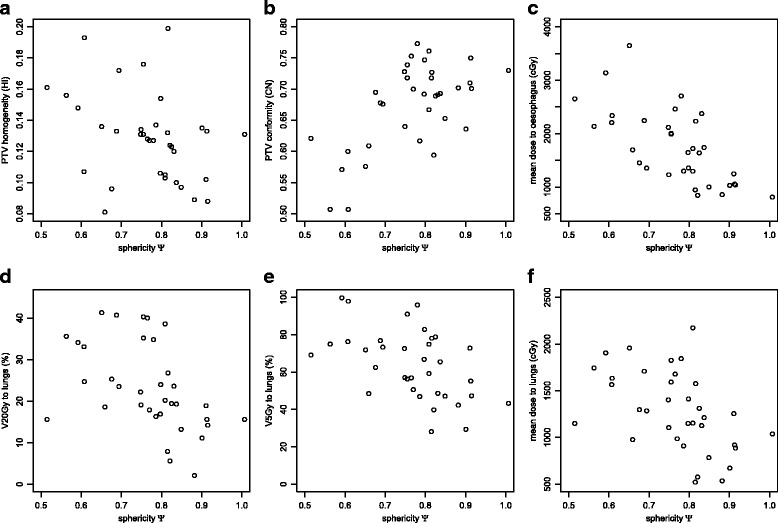
The dependence of the PTV dose homogeneity expressed as HI (**a**), PTV dose conformity CN (**b**), mean dose to the oesophagus (**c**), V20Gy and V5Gy values for lungs (**d**, **e**), and mean dose to the lungs (**f**) on the target volume sphericity *Ψ*. All dosimetric parameters were calculated using DCAT treatment plans. An individual patient case is represented as a point in the diagram; its *x*- and *y*-coordinates represent its relevant parameter values

Figure 6 shows the dependence of several clinical dosimetric parameters obtained by DCAT plans on the target volume sphericity. The dose homogeneity index HI exhibits a moderate and significant (*ρ*=−0.402,*p*=0.02) negative correlation (Fig. 6 a), meaning that the dose distribution is more homogenous in more spherical PTVs. The dose conformity index CN (Fig. 6 b) exhibits moderate and significant (*ρ*=0.463,*p*<0.01) positive correlation. This is expected – when using a conformal technique to treat a highly concave target, the treated volume can be significantly larger than the target. The mean dose to oesophagus (Fig. 6 c) shows a strong and significant negative correlation with *Ψ* (*ρ*=−0.676, *p*<0.01). All three dosimetric parameters for lungs show a moderate and significant negative correlation with sphericity: V20Gy value (Fig. 6 d; *ρ*=−0.573,*p*<0.01), V5Gy value (Fig. 6 e; *ρ*=−0.507, *p*<0.01), and the mean lung dose (Fig. 6 f; *ρ*=−0.558, *p*<0.01). On the other hand, the correlation of D1cc value for the spinal cord is weak and not statistically significant (*ρ*=0.208, *p*=0.23; not shown).

Unlike DCAT plans, hand-crafted conventional 3D CRT plans only show a weak and not statistically significant (*ρ*=−0.265,*p*=0.12; not shown) correlation between *Ψ* and dose homogeneity. In all other aspects, they behave similarly to DCAT plans: they exhibit weak and not statistically significant dependence of D1cc value for the spinal cord on *Ψ*, a strong and statistically significant dependence of the mean dose to oesophagus, and a moderate and statistically significant dependence of CN and all three lung parameters (not shown).

### Target volume location

**Fig. 7:**
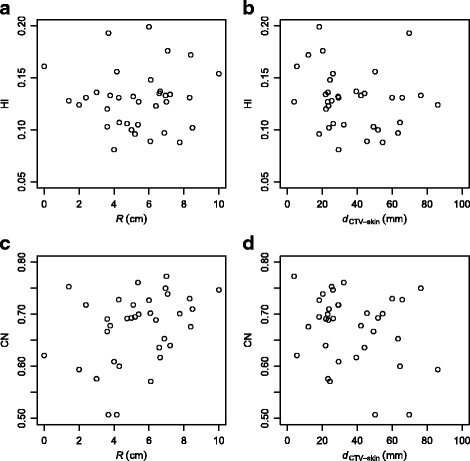
The dependence of the PTV dose homogeneity expressed as HI (**a**, **b**) and PTV dose conformity CN (**c**, **d**) on either the isocentre displacement from the patient origin in the transversal plane and the minimal distance between CTV and external contour. All dosimetric parameters were calculated using DCAT treatment plans. An individual patient case is represented as a point in the diagram; its *x*- and *y*-coordinates represent its relevant parameter values

Figure 7 shows the dependence of the PTV dose homogeneity (HI) and PTV dose conformity (CN) obtained by DCAT plans on either the isocentre displacement from the patient origin *R* or the minimal distance between the CTV and the external contour (*d*_CTV−skin_). The correlation between homogeneity index HI and *R* (Fig. 7 a) is very weak and not statistically significant (*ρ*=0.05, *p*=0.80); the correlation between HI and *d*_CTV−skin_ (Fig. 7 b) is weak and also not statistically significant (*ρ*=−0.24, *p*=0.16). The conformity index CN is weakly yet statistically significantly corretaled with *R* (Fig. 7 c; *ρ*=0.35, *p*=0.04); the correlation between CN and *d*_CTV−skin_ (Fig. 7 d) is very weak and not statistically significant (*ρ*=−0.17, *p*=0.32).

Overall, most dosimetric parameters observed exhibit very weak to weak correlation with both *R* and *d*_CTV−skin_. The only exception is the mean dose to oesophagus, which exhibits moderate and statistically significant correlation with *R* (*ρ*=−0.50, *p*<0.01; not shown). Similar correlation also holds for the conventional 3D CRT plans (*ρ*=−0.53, *p*<0.01; not shown). This can be easily understood, as oesophagus usually lies close to the intersection of the superior-inferior axis and the left-right axis, so a target away from the patient origin is automatically also away from oesophagus.

## Discussion

### Dose calculation in lungs

Being highly heterogeneous in its electron density, the thoracic region presents a challenge for dose calculation algorithms. The Anisotropic Analytical Algorithm (AAA), employed by the dose calculation engine in the Eclipse treatment planning system, is known to overestimate dose in the low density region [18, 19]. This effect is more pronounced in regions with electron density lower than that of realistic lungs, small fields, and high energies. As the clinical examples studied here do not satisfy these conditions, it can be understood that our observations (Table 2) do not confirm the forementioned results. The dose in lungs calculated by AAA does not exhibit significant deviation from its measured value. The only medium with consistent deviation observed was the bone, where the calculated dose value was consistently lower than its measured value, irrespective of the treatment technique (3D CRT vs. DCAT).

### Clinical implications of dosimetric parameters

There are many challenges in delivering radiotherapy to lung cancer patients, one of the most important is ensuring low lung toxicity. Radiation induced lung injury in a form of radiation pneumonitis occurs post-treatment within six months after the treatment and affects patient morbidity and mortality to a considerable degree. Factors predictive of radiation pneumonitis include increased age, smoking history, lower lobe location of tumour, poor performance status, pulmonary disfunction, radiation dose to the lung and the volume of lung irradiated. The use of combined dose-volume metrics (such as V5Gy, V20Gy, or MLD) to evaluate plan safety is now standard practice [20, 21]. The assessment of the radiation pneumonitis risk by the radiation oncologist is therefore a critical aspect of radiation therapy treatment planning. When one-third of the total lung volume is exposed to radiation doses of 20 Gy or more, the risk for symptomatic radiation induced pneumonitis is 10–15%. For V20 below 22%, the risk for radiation pneumonitis is nearly zero. Above a V20Gy of 35%, the risk for radiation pneumonitis rises precipitously. When V20Gy is increased to a value greater than 40%, the risk for radiation induced pneumonitis increases to nearly 40–50% [22, 23].

Our results (Fig. 5 d) show a negative correlation between improvements in V20Gy and V5Gy values for lung: if a DCAT plan brought an improvement over a conventional 3D CRT plan at V20Gy, it fared worse at V5Gy, and vice versa, the few DCAT plans which performed better than their conventional 3D CRT counterparts at V5Gy all did worse than 3D CRT at V20Gy. The relative importance of V20Gy vs. V5Gy (or MLD) as a predictor of radiation pneumonitis might serve as a factor in decision favouring conventional 3D CRT over DCAT or vice versa. However, the available clinical evidence does not yet warrant any such decision.

Overall, treatment planning restriction for lung proved to be the most difficult to meet in this study. However, we need to reiterate that the initial selection of cases eligible for the study excluded the ones in which the total dose on target was high and PTV was close to the spinal cord. The fact that all the plans met the criteria of clinical acceptability concerning the dose to spinal cord can be therefore expected. The very high ratio of treatment plans meeting the criterion for the mean dose to oesophagus is in our view affected by two factors; on the one hand, the threshold value (34 Gy) is quite high in comparison with the total doses used (Table 1), and on the other hand, while 3D CRT plans were created by a skillful dosimetrist/physicist who consciously avoided the structure, DCAT as a rule produces highly conformal dose distribution which compensates for the lack of intelligent guidance.

### Interplay effect in clinical practice

One motivating factor for exploring the possible use of DCAT was the fact that, being a purely conformal technique, it avoids the interplay effect. Despite the early concern about the interplay effect between the MLC movement and intra-fraction organ motion, intensity-modulation techniques like IMRT [24], VMAT [25], helical tomotherapy [26] or even stereotactic body radiotherapy using radical hypofractionation [27] are being increasingly used for treatment of lung cancer and appear to be safe. A dosimetric study on the interplay effect during stereotactic VMAT lung treatment delivery [28] claims that the effects are well within clinically accepted tolerance levels. An overview of the interplay effect [29] agrees that the interplay effects appear to be small (1–2%) in most typical clinical cases. The dominant effect – the blurring of the dose distribution – is independent of the treatment technique, and is not more pronounced in intensity-modulated than in conformal treatment techniques, although it may have a bigger impact on IMRT because of the tendency to reduce target margins when using advanced treatment techniques. All in all, even though in 20 out 35 cases in this study, field-in-field technique was used in 3D CRT, we do not consider this issue as very important, in particular as the treatment fields which do not encompass the whole target projection only contribute a small fraction to the total dose.

### Dosimetric impact of collimator angle

Setting the collimator angle to 45° in DCAT plans may not appear as the optimal choice with respect to the area shielded by MLC only. Indeed in our study, the average opening defined by the jaws at the collimator angle of 45° was 27% larger than the average opening defined by the jaws at the collimator angle of 0° or 90°. Setting the collimator to either 0° or 90° however has its drawbacks too. When using a collimator angle of 0°, one and the same slice of tissue is always exposed to the interleaf leakage, while tilting the collimator results in spreading the leakage dose over a larger volume. For concave target shapes, collimator angle of 90° often results in more MLC travel than other choices. We have shown however that the dosimetric impact of the collimator angle is small. The impact of the collimator angle seems to matter more in VMAT: while the original study argued for a collimator angle set to 45° [30], in some specific cases even the collimator rotated to 90° appears to be advantegous [31].

### Monitor units and treatment time

Lung cancer patients, particularly in the palliative setting, often have bad performance status, and every effort towards shortening the time they spend on the treatment table should be considered worthwhile. As a rule, DCAT treatment plans use fewer monitor units than conventional 3D CRT treatment plans (Fig. 4 d). This alone means a shorter irradiation time for the patient. The total treatment time includes the overhead of patient set-up, which is equal in both the conventional 3D CRT treatment and DCAT treatment and generally exceeds the beam-on time. On the other hand, the time spent due to the required gantry and/or collimator rotation between individual static treatment fields in the conventional 3D CRT increases the difference in the patient door-to-door time even further. All in all, while the ratio of MU spent for both techniques does not directly translate into the ratio of the overall treatment times, a shortening of 1–2 min is to be expected.

## Conclusions

Using film dosimetry, we verified that DCAT is at least as reliable as conventional 3D CRT in terms of dosimetric accuracy. Similar tests are probably necessary at every site wishing to employ the technique, in particular if no tests specific for DCAT have been conducted during the initial machine commissioning.

In most cases, DCAT plans do not pose a dosimetric advantage over manually crafted conventional 3D CRT plans. Even though DCAT can thus not be recommended as a replacement for the conventional 3D CRT, patient anatomy and the treatment prescription may in some cases nevertheless lead to a dosimetrically favourable result with DCAT. Taking into account also a shorter overall treatment time with DCAT, we believe there is a niche for it in radiotherapy. Based on the results of this study, DCAT has been commisioned for use and introduced as a treatment option in our clinic, and is predominantly being used for palliative lung cancer patients.

## Appendix: Target volume sphericity

In general, target volume is a complex 3D shape. For the purpose of this analysis, we will introduce a sphericity parameter which measures the departure of target volume shape from a sphere and does not depend on its size [32]. Sphericity *Ψ* of an object with a given surface area *A* and volume *V* is defined as the surface area of a sphere with a volume equal to the volume of the object, divided by the surface area of the object:

1 $$  \Psi = \frac{\pi^{1/3} (6 V)^{2/3}}{A}.  $$

Sphericity is a dimensionless parameter and can attain values from 0 to 1, with 1 being the value of a perfect sphere. The more an object departs from it, the lower its sphericity is.

While all treatment planning systems provide the volume of a defined structure as parameter, surface area is generally not easily available. We can circumvent this limitation by making use of the onion-like structure of ICRU target volumes and define:

2 $$  \tilde{A} = \frac{V_{\text{PTV}} - V_{\text{CTV}}}{d}, \quad \tilde{V} = \frac{V_{\text{CTV}} + V_{\text{PTV}}}{2}.  $$

Here, *V*_CTV_ and *V*_PTV_ are volumes of CTV and PTV, respectively, and *d* is the safety margin used to construct PTV from CTV. Then, a sphericity-like parameter can be introduced as

3 $$  \tilde{\Psi} = \frac{\pi^{1/3} d \left(3 (V_{\text{CTV}} + V_{\text{PTV}}) \right)^{2/3}} {V_{\text{PTV}} - V_{\text{CTV}}}.  $$

This parameter does not apply to either CTV or PTV, but to a structure in between the two. Overall, $\tilde {\Psi }$ behaves like sphericity, yielding high values for near-spherical shapes and low values for highly concave shapes with many protrusions. The results are the most consistent among the shapes in which the same CTV-PTV margin is applied. Unlike *Ψ*, however, it can exceed the value of 1 in certain cases. Despite its simplicity and limitations, we find it a useful parameter to characterize the target shape.

## Additional file

Additional file 1 Supplementary data. (ZIP 4.96 MB)

## References

[1] Torre LA, Bray F, Siegel RL, Ferlay J, Lortet-Tieulent J, Jemal A (2015). Global cancer statistics, 2012. CA Cancer J Clin.

[2] Cancer Registry of Slovenia (2015). Cancer in Slovenia 2012.

[3] Bortfeld T, Jokivarsi K, Goitein M, Kung J, Jiang SB (2002). Effects of intra-fraction motion on IMRT dose delivery: statistical analysis and simulation. Phys Med Biol.

[4] Jiang SB, Pope C, Al Jarrah KM, Kung JH, Bortfeld T, Chen GT (2003). An experimental investigation on intra-fractional organ motion effects in lung IMRT treatments. Phys Med Biol.

[5] Berbeco RI, Pope CJ, Jiang SB (2006). Measurement of the interplay effect in lung IMRT treatment using EDR2 films. J Appl Clin Med Phys.

[6] Keall PJ, Mageras GS, Balter JM, Emery RS, Forster KM, Jiang SB, Kapatoes JM, Low DA, Murphy MJ, Murray BR (2006). The management of respiratory motion in radiation oncology report of AAPM task group 76a. Med Phys.

[7] Shi C, Tazi A, Fang DX, Iannuzzi C (2013). Implementation and evaluation of modified dynamic conformal arc (MDCA) technique for lung SBRT patients following RTOG protocols. Med Dosim.

[8] Rauschenbach BM, Mackowiak L, Malhotra HK (2014). A dosimetric comparison of three-dimensional conformal radiotherapy, volumetric-modulated arc therapy, and dynamic conformal arc therapy in the treatment of non-small cell lung cancer using stereotactic body radiotherapy. J Appl Clin Med Phys.

[9] Marks LB, Yorke ED, Jackson A, Ten Haken RK, Constine LS, Eisbruch A, Bentzen SM, Nam J, Deasy JO (2010). Use of normal tissue complication probability models in the clinic. Int J Radiat Oncol Biol Phys.

[10] Méndez I, Peterlin P, Hudej R, Strojnik A, Casar B (2014). On multichannel film dosimetry with channel-independent perturbations. Med Phys.

[11] Remeijer P, Geerlof E, Ploeger L, Gilhuijs K, van Herk M, Lebesque JV (2000). 3-D portal image analysis in clinical practice: an evaluation of 2-D and 3-D analysis techniques as applied to 30 prostate cancer patients. Int J Radiat Oncol Biol Phys.

[12] Low DA, Harms WB, Mutic S, Purdy JA (1998). A technique for the quantitative evaluation of dose distributions. Med Phys.

[13] R Core Team (2014). R: A Language and Environment for Statistical Computing.

[14] Thompson RF (2014). RadOnc: an R package for analysis of dose-volume histogram and three-dimensional structural data. J Radiat Oncol Inform.

[15] Van’t Riet A, Mak AC, Moerland MA, Elders LH, van der Zee W (1997). A conformation number to quantify the degree of conformality in brachytherapy and external beam irradiation: application to the prostate. Int J Radiat Oncol Biol Phys.

[16] Wu Q, Mohan R, Morris M, Lauve A, Schmidt-Ullrich R (2003). Simultaneous integrated boost intensity-modulated radiotherapy for locally advanced head-and-neck squamous cell carcinomas. i: dosimetric results. Int J Radiat Oncol Biol Phys.

[17] Yoon M (2007). A new homogeneity index based on the statistical analysis of dose volume histogram. J Appl Clin Med Phys.

[18] Fogliata A, Vanetti E, Albers D, Brink C, Clivio A, Knöös T, Nicolini G, Cozzi L (2007). On the dosimetric behaviour of photon dose calculation algorithms in the presence of simple geometric heterogeneities: comparison with monte carlo calculations. Phys Med Biol.

[19] Kry SF, Alvarez P, Molineu A, Amador C, Galvin J, Followill DS (2013). Algorithms used in heterogeneous dose calculations show systematic differences as measured with the radiological physics center’s anthropomorphic thorax phantom used for RTOG credentialing. Int J Radiat Oncol Biol Phys.

[20] Kwa SL, Lebesque JV, Theuws JC, Marks LB, Munley MT, Bentel G, Oetzel D, Spahn U, Graham MV, Drzymala RE (1998). Radiation pneumonitis as a function of mean lung dose: an analysis of pooled data of 540 patients. Int J Radiat Oncol Biol Phys.

[21] Fay M, Tan A, Fisher R, Mac Manus M, Wirth A, Ball D (2005). Dose-volume histogram analysis as predictor of radiation pneumonitis in primary lung cancer patients treated with radiotherapy. Int J Radiat Oncol Biol Phys.

[22] Marks LB, Bentzen SM, Deasy JO, Bradley JD, Vogelius IS, El Naqa I, Hubbs JL, Lebesque JV, Timmerman RD, Martel MK (2010). Radiation dose–volume effects in the lung. Int J Radiat Oncol Biol Phys.

[23] Palma DA, Senan S, Tsujino K, Barriger RB, Rengan R, Moreno M, Bradley JD, Kim TH, Ramella S, Marks LB (2013). Predicting radiation pneumonitis after chemoradiation therapy for lung cancer: an international individual patient data meta-analysis. Int J Radiat Oncol Biol Phys.

[24] Grills IS, Yan D, Martinez AA, Vicini FA, Wong JW, Kestin LL (2003). Potential for reduced toxicity and dose escalation in the treatment of inoperable non–small-cell lung cancer: A comparison of intensity-modulated radiation therapy (imrt), 3d conformal radiation, and elective nodal irradiation. Int J Radiat Oncol Biol Phys.

[25] Scorsetti M, Navarria P, Mancosu P, Alongi F, Castiglioni S, Cavina R, Cozzi L, Fogliata A, Pentimalli S, Tozzi A (2010). Large volume unresectable locally advanced non-small cell lung cancer: acute toxicity and initial outcome results with rapid arc. Radiat Oncol.

[26] Kron T, Grigorov G, Yu E, Yartsev S, Chen JZ, Wong E, Rodrigues G, Trenka K, Coad T, Bauman G (2004). Planning evaluation of radiotherapy for complex lung cancer cases using helical tomotherapy. Phys Med Biol.

[27] Navarria P, Ascolese AM, Tomatis S, Cozzi L, De Rose F, Mancosu P, Alongi F, Clerici E, Lobefalo F, Tozzi A (2014). Stereotactic body radiotherapy (sbrt) in lung oligometastatic patients: role of local treatments. Radiat Oncol.

[28] Ong C, Verbakel WF, Cuijpers JP, Slotman BJ, Senan S (2011). Dosimetric impact of interplay effect on rapidarc lung stereotactic treatment delivery. Int J Radiat Oncol Biol Phys.

[29] Bortfeld T, Jiang SB, Rietzel E (2004). Effects of motion on the total dose distribution. Semin Radiat Oncol.

[30] Otto K (2008). Volumetric modulated arc therapy: IMRT in a single gantry arc. Med Phys.

[31] Mancosu P, Cozzi L, Fogliata A, Lattuada P, Reggiori G, Cantone MC, Navarria P, Scorsetti M (2010). Collimator angle influence on dose distribution optimization for vertebral metastases using volumetric modulated arc therapy. Med Phys.

[32] Wadell H (1935). Volume, shape, and roundness of quartz particles. J Geol.

