# Supplementary material for: Treating lung cancer with dynamic conformal arc therapy: a dosimetric study
Source: Radiat Oncol. 2017 Jun 2;12:93. doi: 10.1186/s13014-017-0823-y (PMC5457634; doi:10.1186/s13014-017-0823-y)
Supplement: Additional file 1 — Supplementary data. (ZIP 4.96 MB) [file 13014_2017_823_MOESM1_ESM.zip › Additional Files/dataset.pdf]

Primož Peterlin, Karmen Stanič, Ignasi Méndez, Andrej Strojnik (2017). Treating lung cancer with dynamic conformal arc therapy: a dosimetric study. Radiation Oncology
doi:10.1186/s13014-017-0823-y

Supplementary data

Dose volume histograms

Cumulative dose volume histograms (DVHs) for the 35 patient cases in this study are presented separately for each treatment plan (conventional 3D CRT and DCAT). The colors used in DVH for denoting a given structure conform to the pallette below. Note that not all structures were contoured in every case.

GTV	dark red
CTV	red
PTV	blue
lung	magenta
heart	cyan
spinal cord	yellow
spinal cord PORV	dark yellow
esophagus	maroon
liver	green

In the table, the volume is given for each structure, as well as several dosimetric parameters (all in cGy): minimum and maximum point dose contained within the structure, median dose to the structure, mean dose to the structure, and the standard deviation of dose to the structure.

Case 1

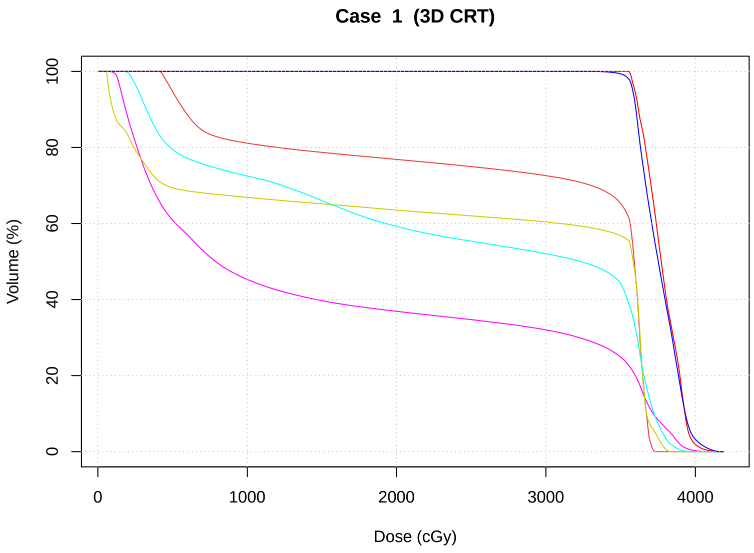

Structure	V [cm3]	Dmin	Dmax	Dmedian	Dmean	Dstd
CTV	138.9	3548.5	4194.9	3771.9	3777.7	116.7
lung	2794.7	61.8	4109.8	788.4	1617.0	1481.6
esophagus	28.3	407.5	3735.0	3591.2	2891.7	1212.0
spinal cord	27.4	56.2	3839.1	3584.3	2393.8	1581.8
heart	725.9	153.0	3975.4	3232.2	2373.8	1405.3
PTV	533.8	3270.2	4194.9	3751.9	3761.5	130.8

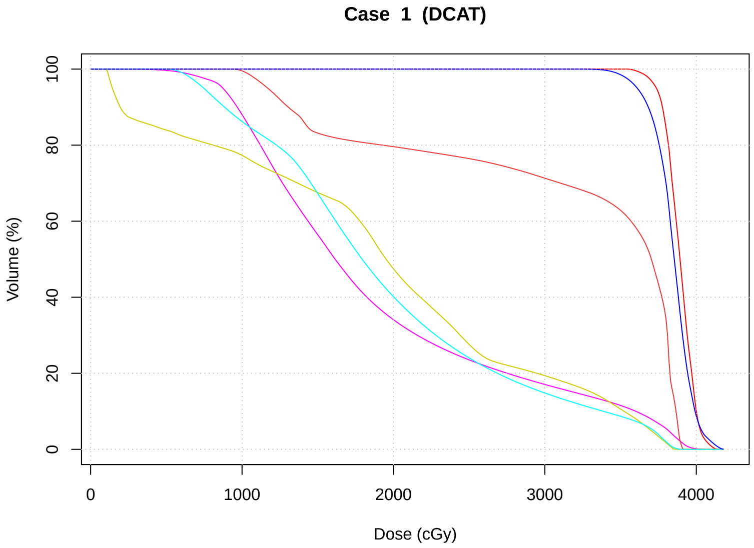

Structure	V [cm3]	Dmin	Dmax	Dmedian	Dmean	Dstd
CTV	138.9	3534.6	4140.5	3892.6	3889.2	91.7
lung	2794.7	206.5	4088.0	1615.1	1905.0	926.1
esophagus	28.3	938.5	3916.2	3703.5	3138.4	985.5
spinal cord	27.4	103.0	3861.8	1951.5	1913.8	1091.2
heart	725.9	495.1	3908.3	1793.4	1957.0	860.1
PTV	533.8	3190.1	4186.0	3854.6	3845.3	127.4

Case 2

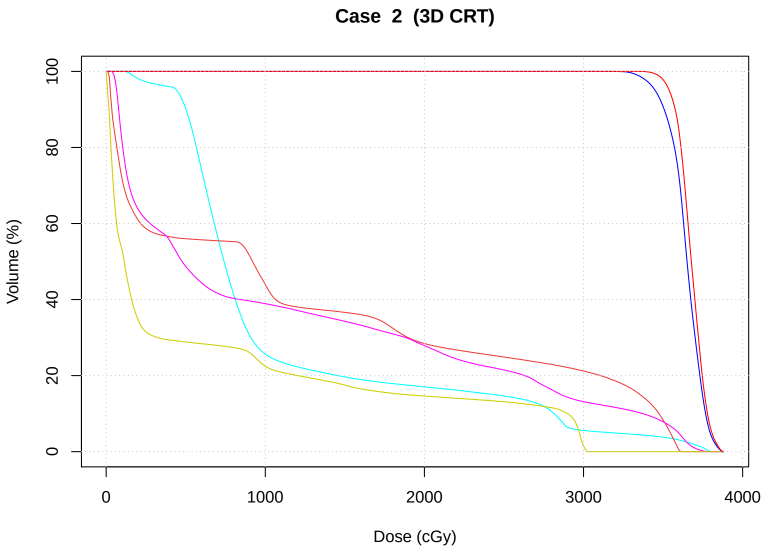

Structure	V [cm3]	Dmin	Dmax	Dmedian	Dmean	Dstd
heart	546.9	108.5	3830.1	741.1	1112.6	898.6
esophagus	35.5	11.9	3614.2	920.2	1286.5	1322.8
lung	4993.9	27.3	3824.2	477.2	1158.2	1248.0
spinal cord	54.1	0.0	3028.5	113.4	641.8	1001.3
PTV	519.1	3112.0	3882.6	3650.5	3644.0	103.4
CTV	323.4	3352.9	3882.6	3677.5	3677.0	80.7

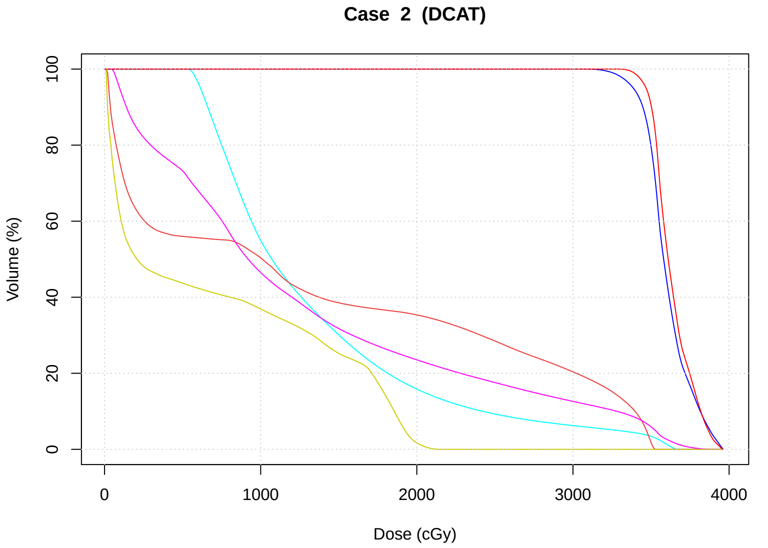

Structure	V [cm3]	Dmin	Dmax	Dmedian	Dmean	Dstd
heart	546.9	506.7	3675.0	1071.9	1342.1	757.5
esophagus	35.5	17.0	3529.8	1010.4	1356.1	1318.9
lung	4993.9	47.2	3910.5	919.9	1285.4	1083.1
spinal cord	54.1	10.4	2148.7	208.1	711.2	756.0
PTV	519.1	3011.4	3967.1	3580.8	3602.7	143.5
CTV	323.4	3274.5	3964.3	3610.0	3634.1	121.9

Case 3

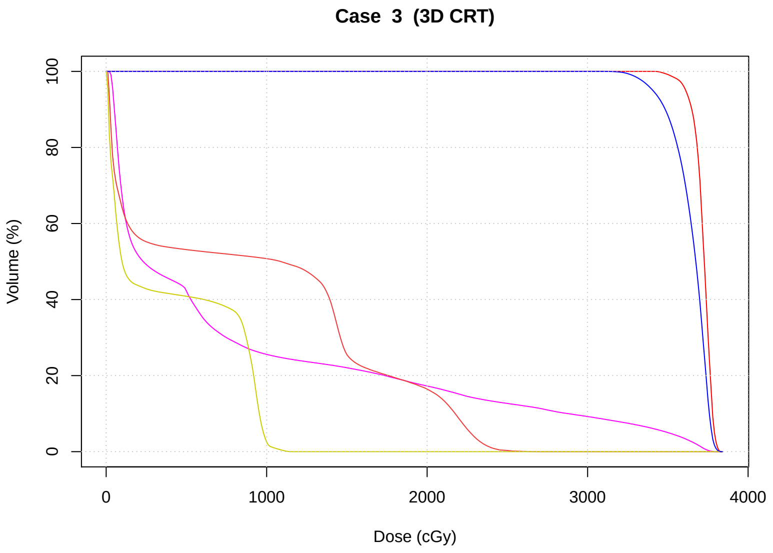

Structure	V [cm3]	Dmin	Dmax	Dmedian	Dmean	Dstd
CTV	96.9	3412.9	3835.0	3727.0	3718.5	57.0
lung	5265.4	21.1	3830.5	230.7	830.8	1103.8
esophagus	38.9	13.8	2820.6	1087.9	919.1	855.0
spinal cord	59.2	0.0	1158.3	98.6	405.0	419.9
PTV	320.4	3070.4	3849.7	3674.1	3643.9	114.3

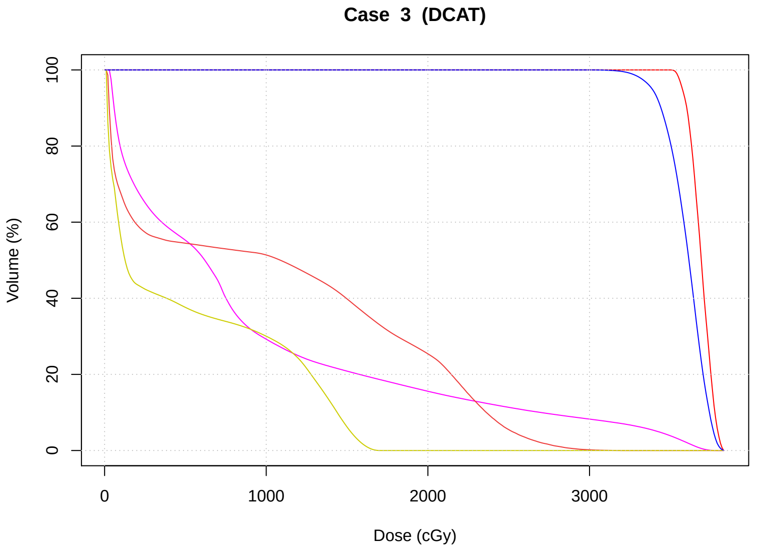

Structure	V [cm3]	Dmin	Dmax	Dmedian	Dmean	Dstd
CTV	96.9	3499.9	3831.8	3691.6	3689.8	65.8
lung	5265.4	25.5	3802.7	619.7	918.8	1015.6
esophagus	38.9	17.3	3221.2	1087.3	1054.8	970.2
spinal cord	59.2	9.7	1702.7	126.5	518.7	586.1
PTV	320.4	3009.1	3831.8	3615.0	3600.0	118.3

Case 4

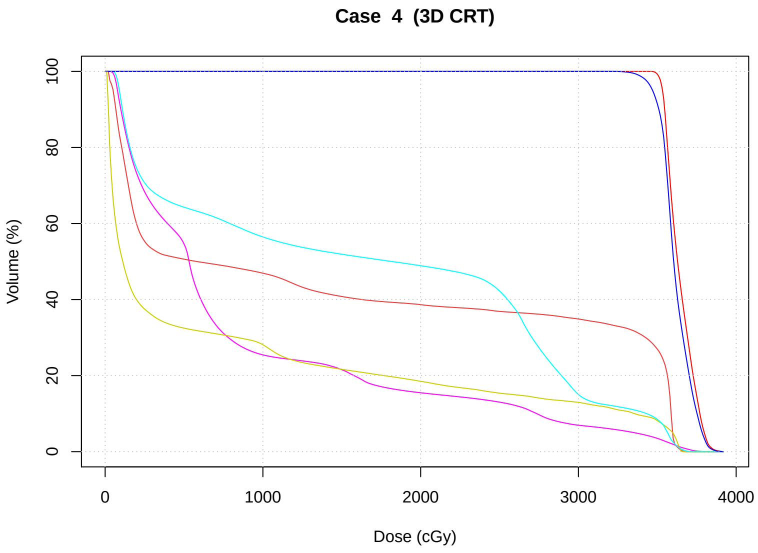

Structure	V [cm3]	Dmin	Dmax	Dmedian	Dmean	Dstd
CTV	285.8	3434.9	3922.7	3628.5	3644.7	85.1
lung	3778.3	28.5	3865.3	532.2	896.0	1002.5
esophagus	39.3	19.1	3681.8	582.0	1534.3	1557.3
spinal cord	52.9	6.4	3656.4	111.4	812.7	1218.3
heart	585.1	51.7	3717.3	1821.4	1648.0	1304.3
PTV	516.9	3186.7	3922.7	3603.8	3617.4	95.2

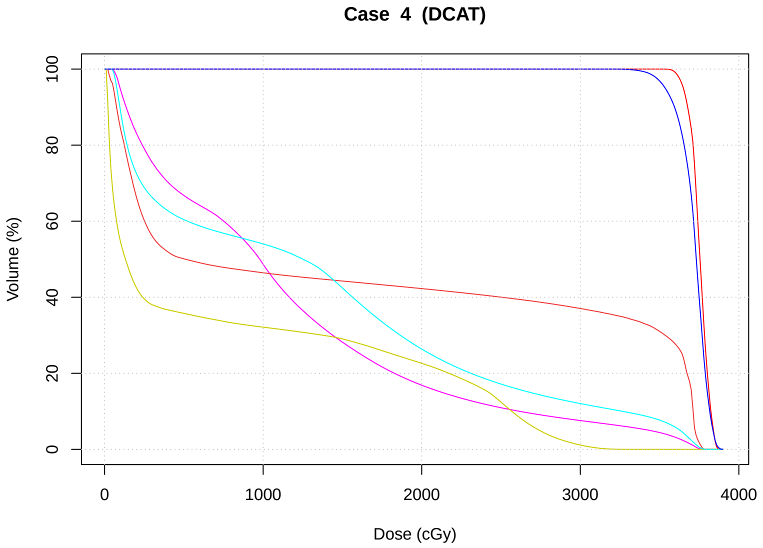

Structure	V [cm3]	Dmin	Dmax	Dmedian	Dmean	Dstd
CTV	285.8	3508.0	3879.2	3755.0	3752.4	55.9
lung	3778.3	26.4	3822.6	978.2	1150.1	967.4
esophagus	39.3	22.5	3784.5	509.5	1646.2	1621.8
spinal cord	52.9	9.9	3267.0	131.5	806.7	1046.3
heart	585.1	44.2	3796.3	1250.9	1313.5	1159.7
PTV	516.9	3179.7	3906.1	3731.6	3716.5	91.0

Case 5

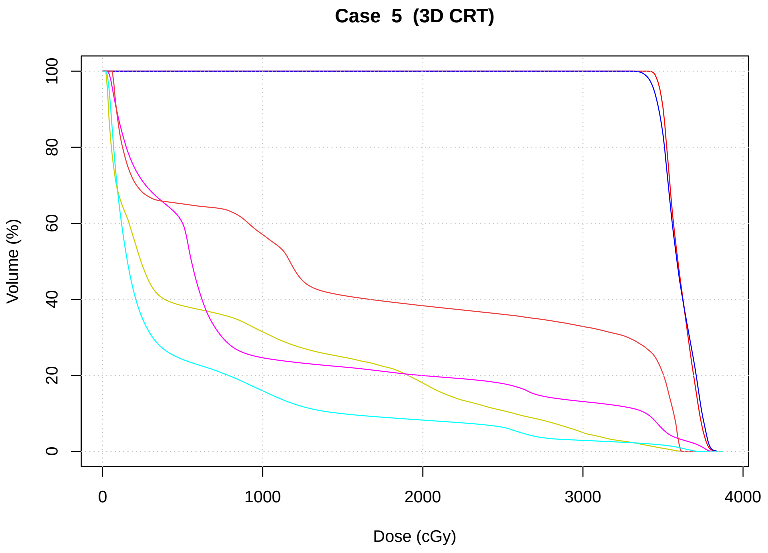

Structure	V [cm3]	Dmin	Dmax	Dmedian	Dmean	Dstd
CTV	335.6	3397.0	3849.9	3593.2	3604.7	86.3
PTV	617.6	3295.9	3879.4	3587.5	3600.0	102.4
spinal cord	30.6	24.0	3625.1	238.8	823.5	1020.1
lung	2829.1	25.2	3862.4	554.5	1002.3	1150.1
esophagus	35.3	60.8	3617.1	1168.9	1638.9	1432.9
heart	837.1	20.2	3737.0	154.3	506.6	805.1

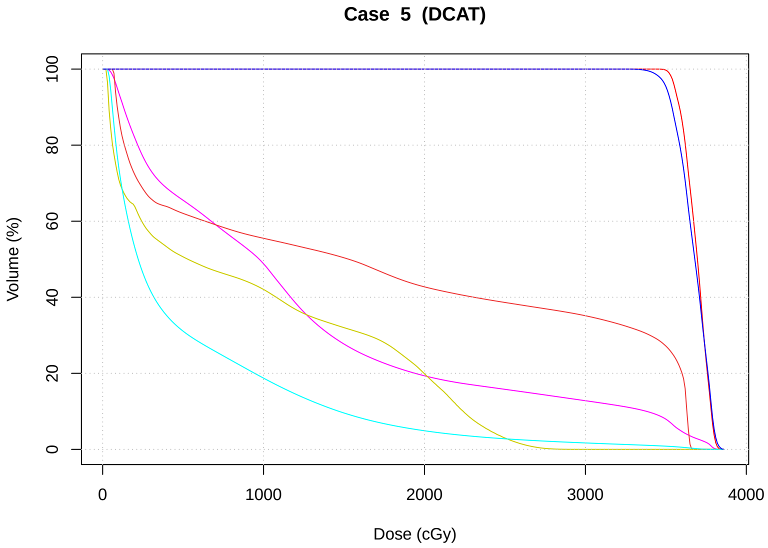

Structure	V [cm3]	Dmin	Dmax	Dmedian	Dmean	Dstd
lung	2829.1	21.6	3839.4	973.0	1212.2	1099.2
CTV	335.6	3443.6	3849.2	3696.2	3688.4	73.7
esophagus	35.3	65.2	3664.0	1528.8	1740.4	1492.4
spinal cord	30.6	25.1	2965.8	529.1	923.4	893.5
heart	837.1	27.8	3771.4	220.7	534.7	691.4
PTV	617.6	3245.7	3861.2	3679.9	3670.2	95.4

Case 6

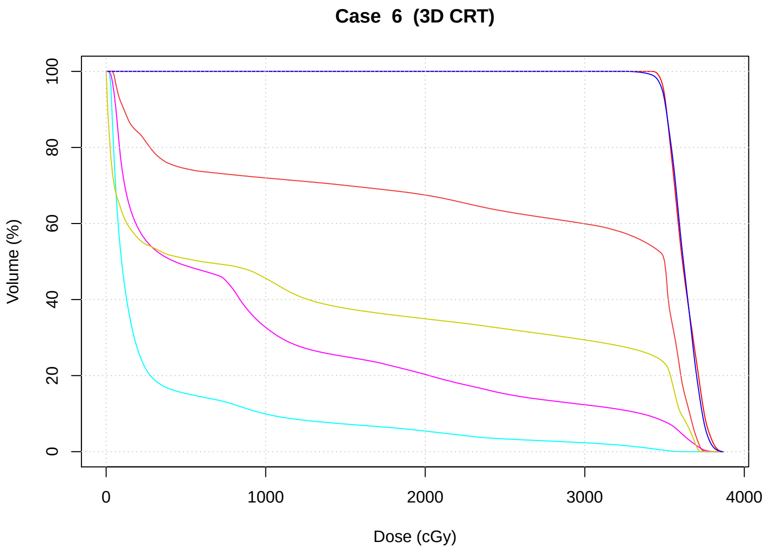

Structure	V [cm3]	Dmin	Dmax	Dmedian	Dmean	Dstd
heart	581.2	22.7	3607.0	96.9	349.7	680.0
CTV	600.6	3421.1	3872.5	3612.2	3623.7	90.1
lung	4319.4	8.0	3839.0	429.3	1000.4	1174.6
esophagus	27.4	44.4	3769.3	3500.6	2446.8	1476.9
spinal cord	49.7	0.0	3732.3	597.6	1402.5	1525.5
PTV	1129.0	3228.3	3872.5	3618.5	3620.9	87.6

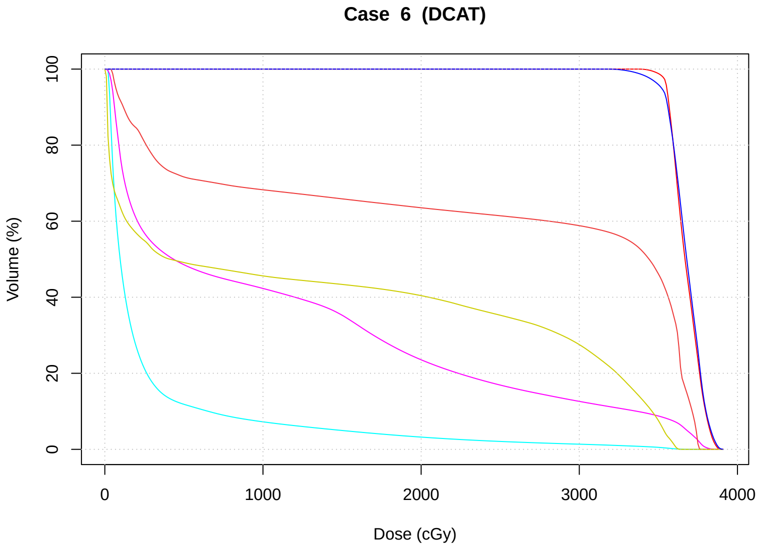

Structure	V [cm3]	Dmin	Dmax	Dmedian	Dmean	Dstd
heart	581.2	21.0	3677.3	96.9	285.4	564.6
CTV	600.6	3376.0	3904.1	3668.4	3676.5	87.8
lung	4319.4	8.5	3877.9	434.8	1128.0	1227.9
esophagus	27.4	42.0	3766.0	3441.3	2376.3	1536.4
spinal cord	49.7	8.5	3639.2	412.0	1407.2	1469.7
PTV	1129.0	3175.5	3914.7	3679.4	3676.9	101.8

Case 7

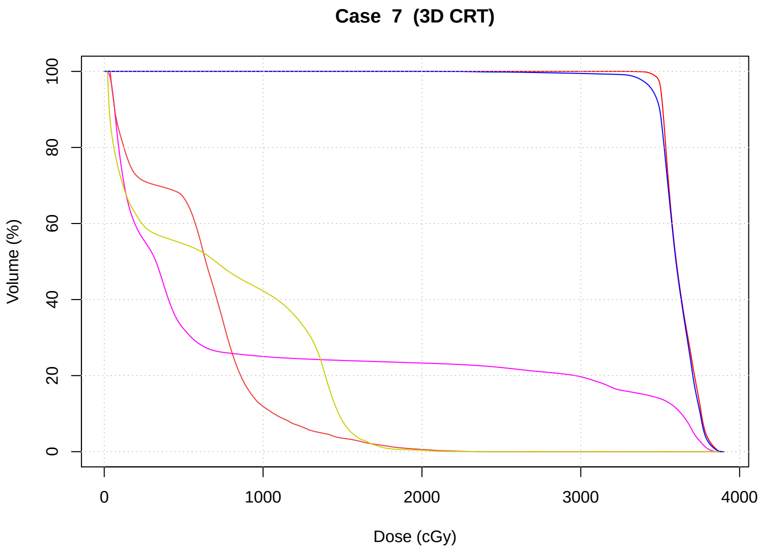

Structure	V [cm3]	Dmin	Dmax	Dmedian	Dmean	Dstd
CTV	396.0	3246.5	3882.5	3601.5	3621.8	93.7
lung	2713.0	12.0	3894.1	325.6	998.9	1359.0
esophagus	30.0	36.0	2430.7	639.3	604.4	415.6
spinal cord	44.1	19.6	2362.8	700.9	727.4	610.4
PTV	873.0	1960.8	3901.5	3599.3	3605.2	127.2

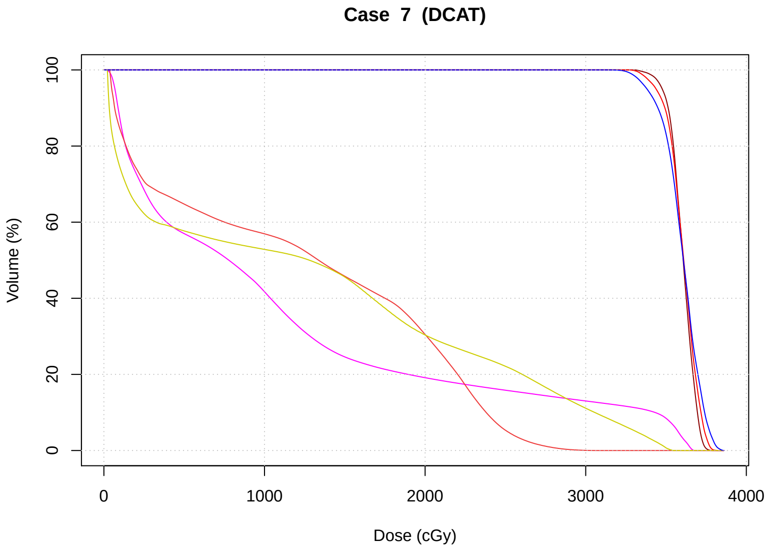

Structure	V [cm3]	Dmin	Dmax	Dmedian	Dmean	Dstd
GTV	171.7	3297.8	3800.9	3606.9	3603.4	73.2
CTV	396.0	3266.8	3831.1	3610.3	3605.0	90.3
lung	2713.0	14.1	3690.0	778.1	1105.9	1133.0
esophagus	30.0	35.3	3063.3	1336.8	1231.1	926.5
spinal cord	44.1	20.7	3549.2	1279.4	1293.8	1182.7
PTV	873.0	3129.4	3860.8	3608.3	3600.0	114.5

Case 8

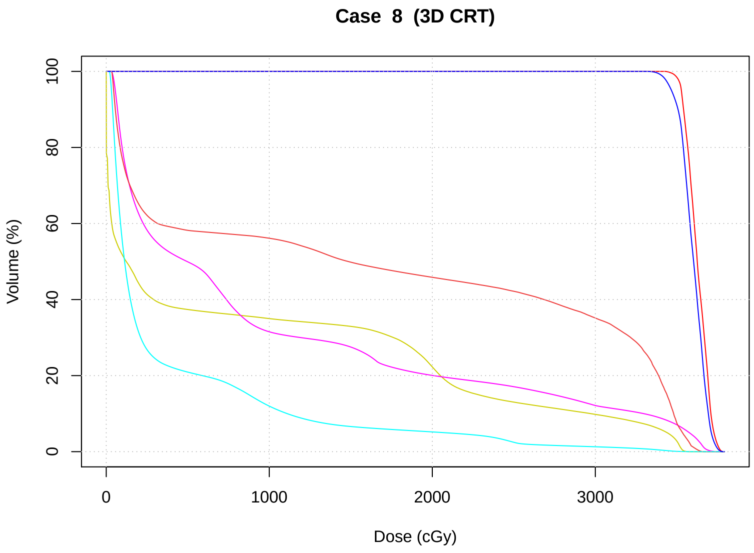

Structure	V [cm3]	Dmin	Dmax	Dmedian	Dmean	Dstd
spinal cord	60.7	0.0	3584.1	122.8	898.5	1188.3
CTV	174.0	3424.1	3792.1	3625.3	3626.9	64.1
lung	2281.2	22.8	3770.2	496.3	1014.8	1173.2
esophagus	33.6	34.3	3669.3	1485.1	1683.8	1476.0
heart	810.3	17.2	3609.1	112.7	385.9	646.1
PTV	448.6	3295.7	3792.1	3602.9	3600.0	74.5

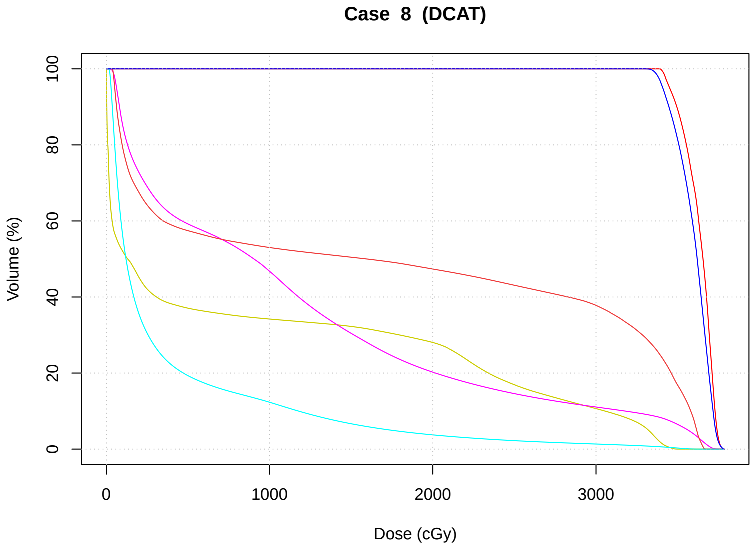

Structure	V [cm3]	Dmin	Dmax	Dmedian	Dmean	Dstd
spinal cord	60.7	0.6	3503.2	131.1	934.9	1224.8
CTV	174.0	3390.5	3782.6	3655.4	3632.7	88.7
lung	2281.2	20.4	3768.9	904.4	1153.2	1104.5
esophagus	33.6	38.1	3670.1	1595.8	1721.2	1521.1
heart	810.3	12.7	3631.2	120.6	377.1	619.4
PTV	448.6	3299.1	3785.4	3619.8	3600.0	100.0

Case 9

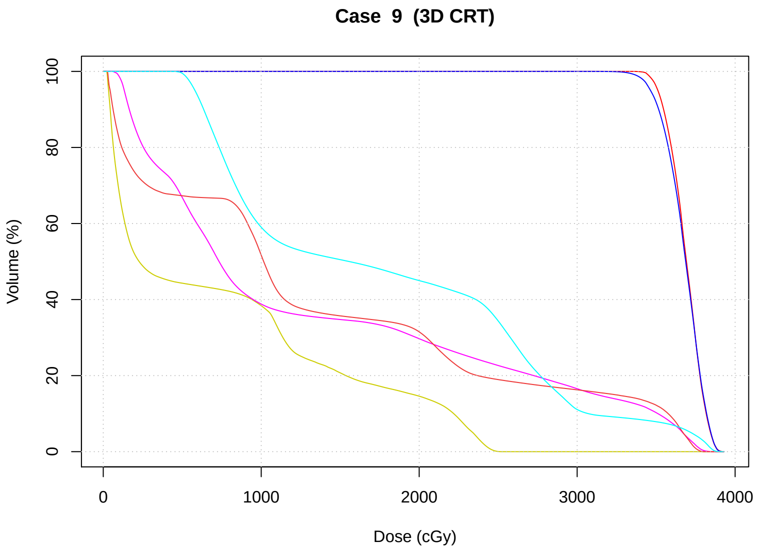

Structure	V [cm3]	Dmin	Dmax	Dmedian	Dmean	Dstd
CTV	606.5	3348.6	3929.4	3691.8	3686.8	101.1
PTV	968.2	2961.4	3929.4	3687.0	3675.9	116.4
spinal cord	59.7	18.4	2516.9	227.1	758.4	817.1
lung	3798.8	55.1	3887.8	733.2	1328.2	1225.3
esophagus	49.6	26.2	3800.1	1015.6	1364.1	1221.0
heart	980.1	437.6	3911.1	1556.7	1779.8	1047.3

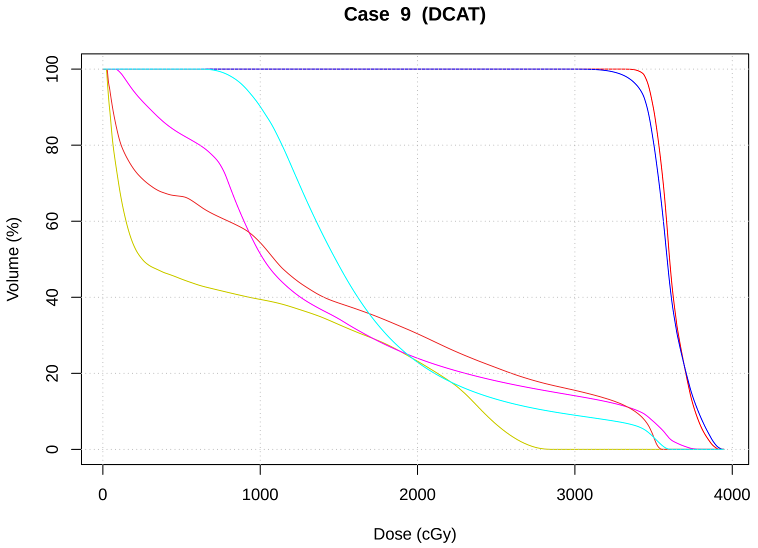

Structure	V [cm3]	Dmin	Dmax	Dmedian	Dmean	Dstd
CTV	606.5	3287.6	3931.4	3602.0	3618.1	101.4
PTV	968.2	2935.9	3947.5	3586.5	3600.0	129.3
spinal cord	59.7	20.9	2849.2	250.1	910.3	970.9
lung	3798.8	80.5	3852.7	1021.0	1412.7	1038.7
esophagus	49.6	26.4	3557.5	1089.4	1357.3	1182.9
heart	980.1	592.4	3617.2	1478.7	1683.3	705.9

Case 10

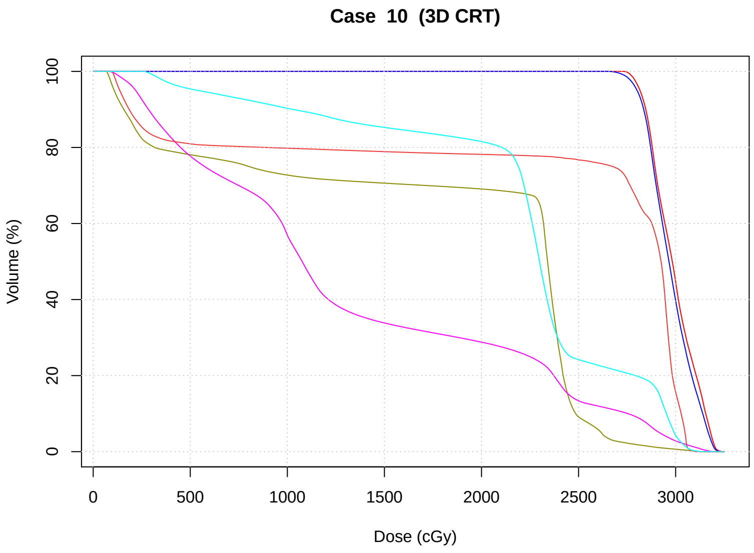

Structure	V [cm3]	Dmin	Dmax	Dmedian	Dmean	Dstd
CTV	370.5	2723.7	3251.4	2982.7	2988.2	114.4
PTV	984.4	2644.4	3251.4	2965.7	2973.5	113.5
spinal cord PORV	156.9	67.1	3137.5	2340.4	1798.1	952.8
lung	3297.4	73.3	3222.2	1075.9	1329.8	889.3
esophagus	37.7	96.3	3112.1	2924.8	2349.6	1090.4
heart	834.2	258.6	3171.5	2298.8	2196.0	670.3

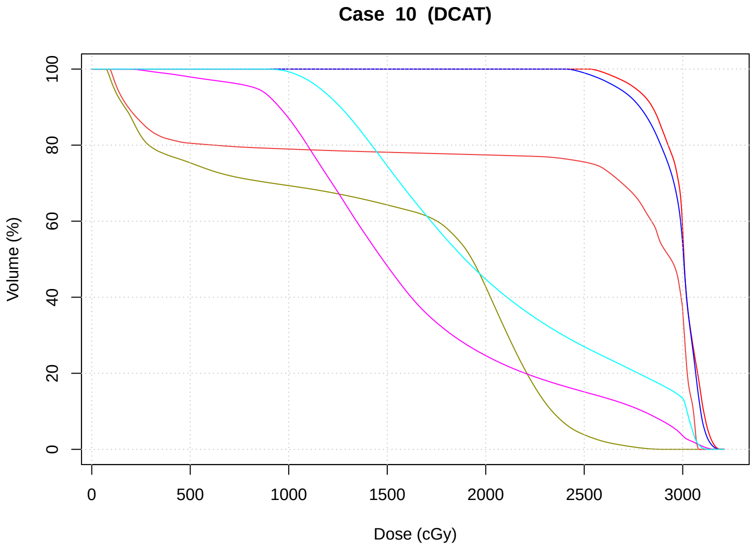

Structure	V [cm3]	Dmin	Dmax	Dmedian	Dmean	Dstd
CTV	370.5	2524.7	3211.9	3006.9	2990.3	110.1
PTV	984.4	2398.1	3211.9	3005.2	2968.3	133.3
spinal cord PORV	156.9	73.5	2896.8	1930.2	1510.9	853.6
lung	3297.4	180.2	3191.2	1475.6	1633.7	662.3
esophagus	37.7	89.2	3082.5	2940.0	2338.8	1108.3
heart	834.2	867.6	3132.2	1894.7	2023.8	632.9

Case 11

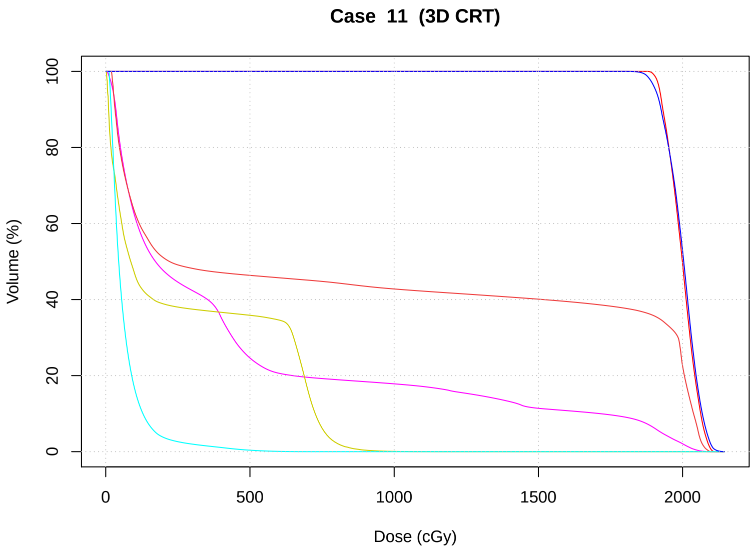

Structure	V [cm3]	Dmin	Dmax	Dmedian	Dmean	Dstd
CTV	377.9	1875.1	2123.1	1999.3	1998.3	47.9
lung	3275.1	3.5	2106.0	172.3	466.6	603.1
esophagus	38.0	19.5	2097.3	221.3	906.0	913.8
spinal cord	59.0	1.7	1171.3	86.7	291.1	315.2
heart	599.4	9.7	699.8	44.9	66.0	69.0
PTV	661.3	1801.7	2145.3	2004.0	2000.0	53.8

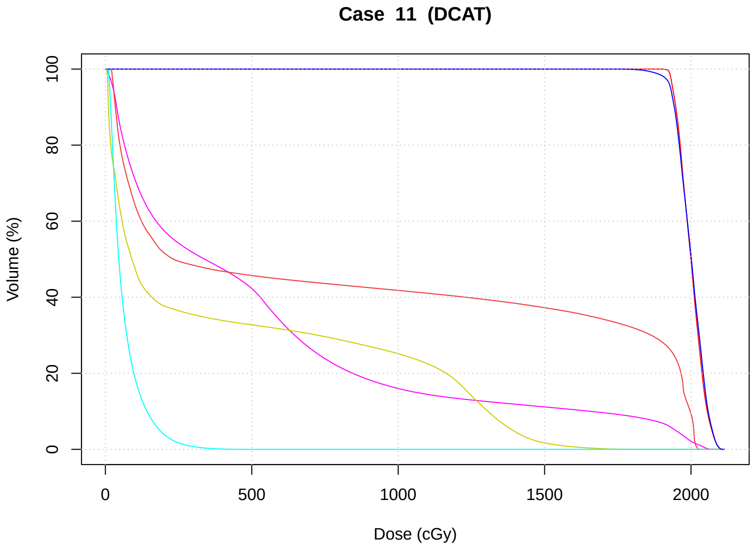

Structure	V [cm3]	Dmin	Dmax	Dmedian	Dmean	Dstd
CTV	377.9	1894.7	2113.3	1999.8	2000.8	40.4
lung	3275.1	2.9	2091.8	339.1	536.0	579.3
esophagus	38.0	19.7	2027.1	232.7	857.6	868.2
spinal cord	59.0	3.9	1815.3	90.6	429.6	537.1
heart	599.4	8.1	472.0	45.9	65.8	57.5
PTV	661.3	1740.0	2114.2	2001.4	2000.0	46.1

Case 12

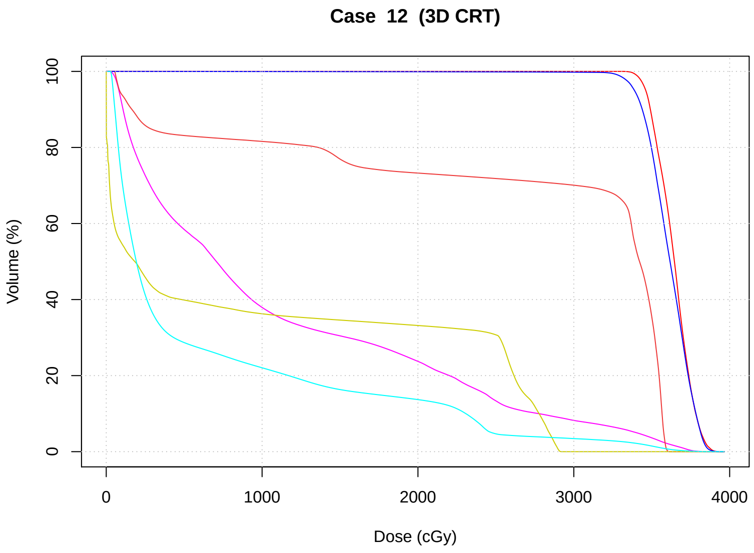

Structure	V [cm3]	Dmin	Dmax	Dmedian	Dmean	Dstd
CTV	139.7	3314.5	3929.2	3645.6	3640.0	109.4
lung	5413.8	19.3	3934.0	706.6	1106.8	1062.7
esophagus	35.8	53.5	3612.2	3421.7	2677.1	1305.1
spinal cord	84.7	0.0	2924.3	183.1	1001.4	1212.2
PTV	641.9	0.0	3966.6	3617.5	3609.8	154.0
heart	802.6	26.6	3856.7	192.9	636.6	900.0

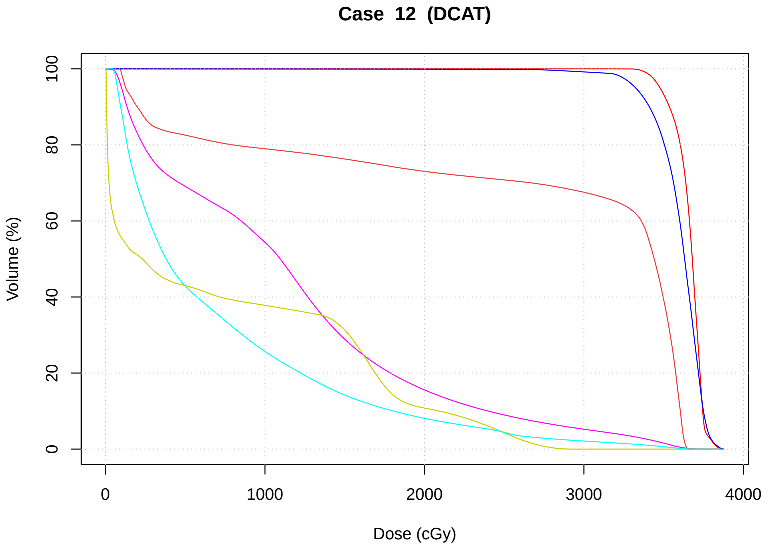

Structure	V [cm3]	Dmin	Dmax	Dmedian	Dmean	Dstd
CTV	139.7	3118.6	3869.7	3679.4	3660.7	87.0
lung	5413.8	18.9	3757.3	1095.7	1150.1	885.6
esophagus	35.8	91.9	3658.0	3440.9	2652.8	1326.6
spinal cord	84.7	2.7	2924.8	231.8	773.7	885.5
PTV	641.9	5.0	3876.6	3631.5	3600.0	166.7
heart	802.6	46.7	3692.4	380.6	711.4	760.2

Case 13

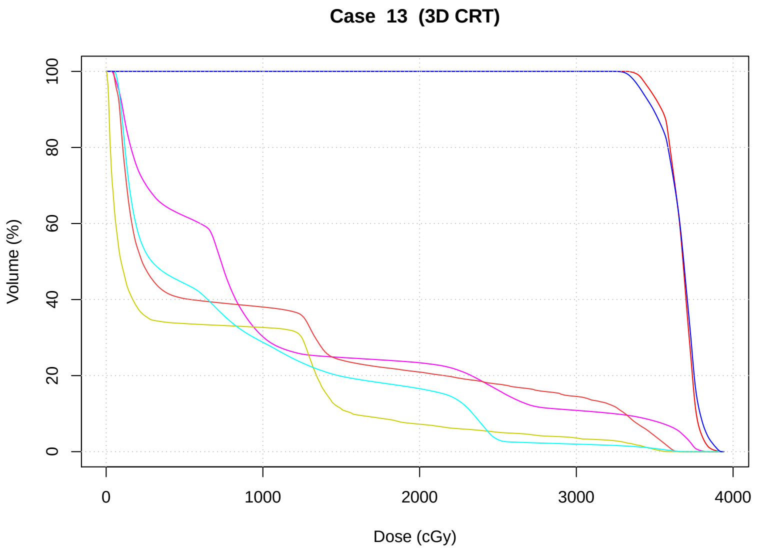

Structure	V [cm3]	Dmin	Dmax	Dmedian	Dmean	Dstd
CTV	581.5	3321.8	3934.5	3680.2	3665.7	93.6
lung	4344.2	33.4	3884.5	733.0	1098.1	1131.8
esophagus	31.7	44.2	3672.9	229.0	998.3	1202.1
spinal cord	87.3	0.0	3589.6	95.0	616.8	868.4
heart	413.9	44.5	3701.2	292.0	784.3	876.3
PTV	1043.9	3213.6	3941.9	3685.3	3664.0	117.6

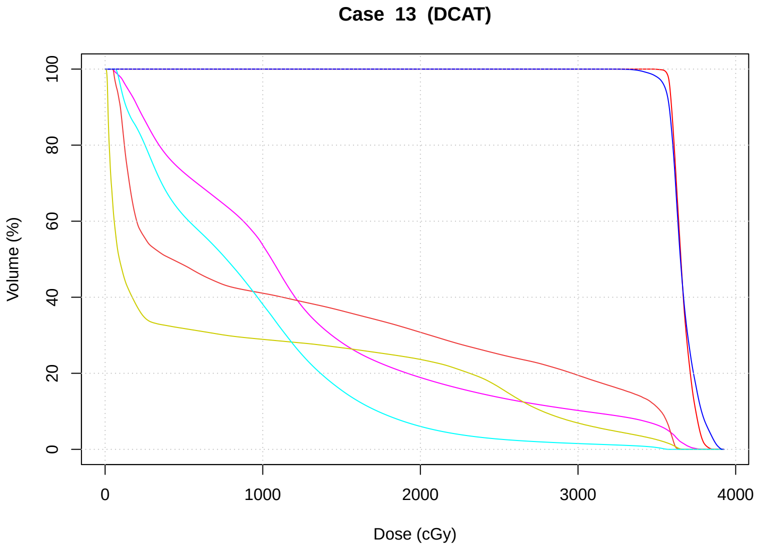

Structure	V [cm3]	Dmin	Dmax	Dmedian	Dmean	Dstd
CTV	581.5	3465.4	3861.0	3652.8	3661.6	58.3
lung	4344.2	31.3	3827.4	1056.2	1255.4	996.9
esophagus	31.7	47.2	3641.6	424.8	1248.2	1338.1
spinal cord	87.3	6.7	3667.5	91.0	811.3	1169.3
heart	413.9	59.4	3575.3	769.9	869.8	677.5
PTV	1043.9	3212.5	3925.8	3649.6	3664.2	85.5

Case 14

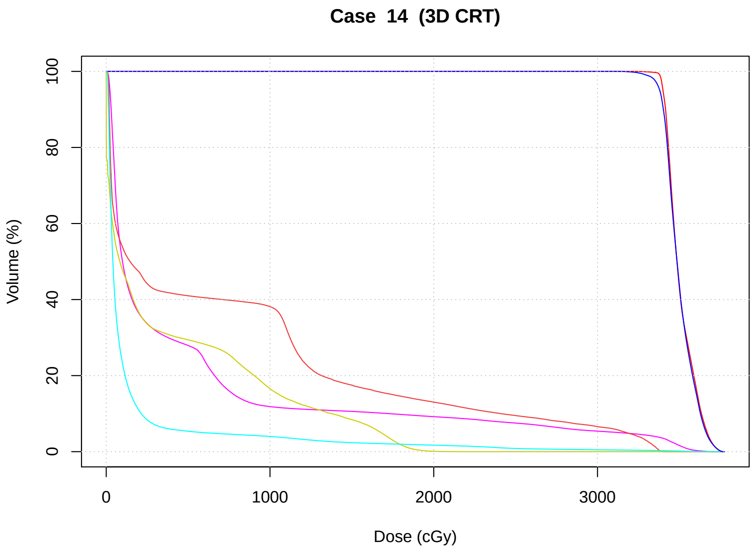

Structure	V [cm3]	Dmin	Dmax	Dmedian	Dmean	Dstd
CTV	331.6	3231.5	3775.2	3485.1	3506.9	84.8
lung	4225.6	0.0	3734.8	99.2	500.7	882.1
esophagus	43.7	5.7	3406.4	145.6	757.3	985.3
spinal cord	74.8	0.0	2254.0	83.2	395.1	554.9
heart	1209.8	4.6	3676.0	40.4	148.0	408.1
PTV	535.6	3101.1	3775.2	3485.7	3500.0	89.8

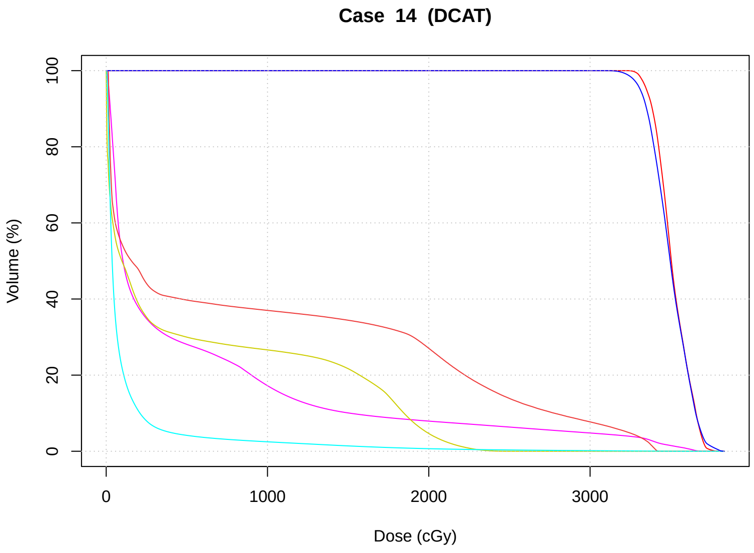

Structure	V [cm3]	Dmin	Dmax	Dmedian	Dmean	Dstd
CTV	331.6	3235.0	3797.0	3503.7	3513.0	101.3
lung	4225.6	1.9	3723.5	104.0	521.7	856.3
esophagus	43.7	12.0	3426.3	156.8	946.5	1163.6
spinal cord	74.8	0.2	2483.1	97.4	542.8	754.6
heart	1209.8	2.7	3582.8	36.9	116.3	300.4
PTV	535.6	3086.1	3833.0	3496.5	3500.0	117.8

Case 15

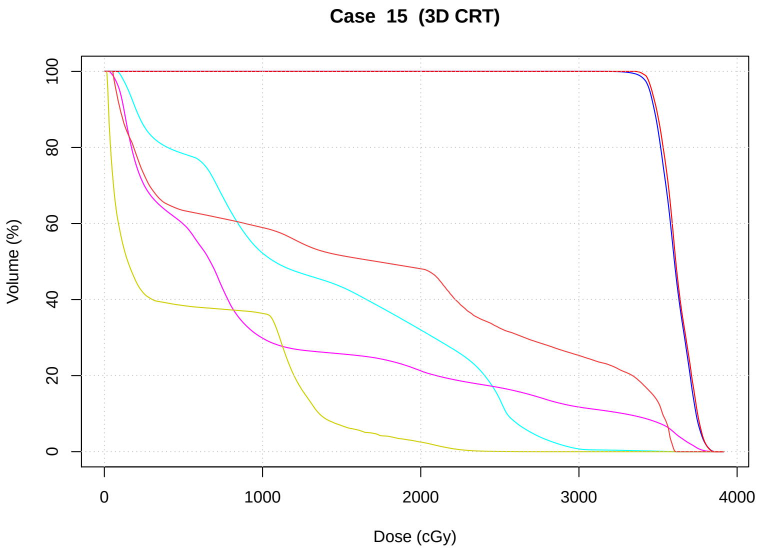

Structure	V [cm3]	Dmin	Dmax	Dmedian	Dmean	Dstd
heart	439.9	77.4	3625.3	1074.7	1349.2	900.3
PTV	748.8	3102.7	3895.2	3603.6	3606.9	101.2
lung	3667.8	29.1	3916.8	669.4	1080.1	1134.9
spinal cord	75.9	11.4	2746.2	148.5	555.6	626.1
CTV	354.7	3356.8	3860.3	3612.2	3618.5	96.4
esophagus	33.4	51.7	3615.5	1723.1	1670.2	1342.1

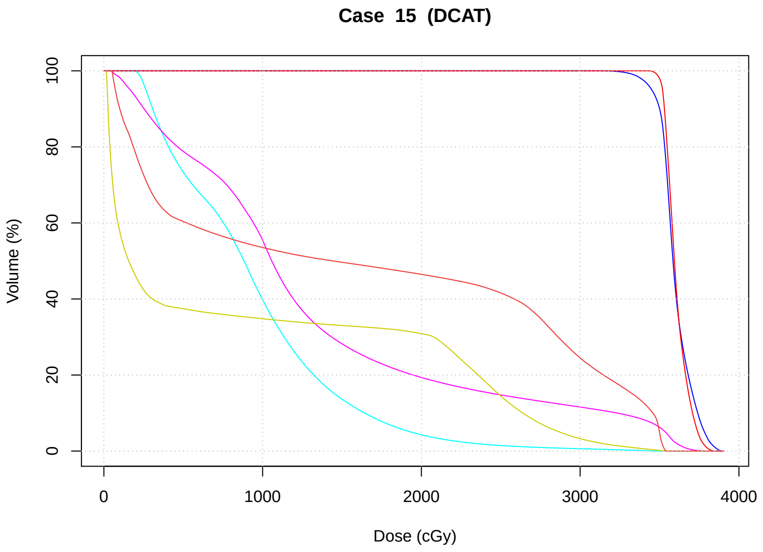

Structure	V [cm3]	Dmin	Dmax	Dmedian	Dmean	Dstd
heart	439.9	183.4	3478.0	883.0	934.4	545.3
PTV	748.8	3092.3	3905.9	3584.8	3600.0	99.6
lung	3667.8	32.6	3800.0	1057.6	1311.5	984.5
spinal cord	75.9	14.5	3583.1	156.4	922.6	1135.9
CTV	354.7	3413.1	3845.9	3593.9	3606.9	68.8
esophagus	33.4	50.4	3553.7	1443.3	1639.3	1365.5

Case 16

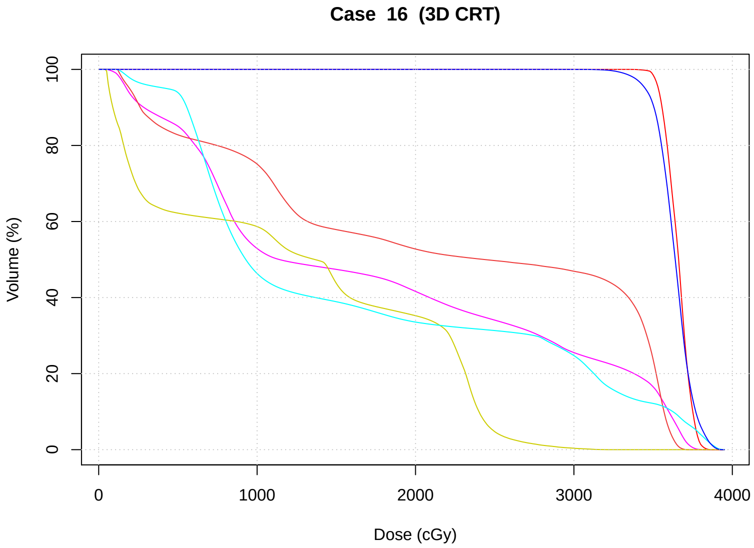

Structure	V [cm3]	Dmin	Dmax	Dmedian	Dmean	Dstd
CTV	343.5	3347.8	3892.0	3661.0	3655.4	73.4
lung	2331.0	51.3	3880.5	1140.2	1749.0	1262.1
esophagus	30.5	117.5	3719.4	2435.7	2173.5	1331.4
spinal cord	29.8	47.6	3215.0	1364.9	1253.2	959.2
heart	581.7	114.0	3950.2	928.4	1634.4	1216.2
PTV	779.8	2863.6	3948.2	3639.4	3635.0	109.9

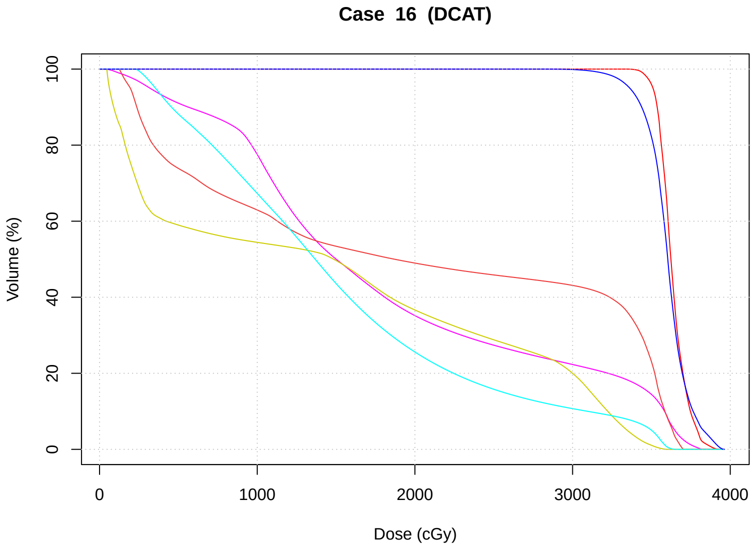

Structure	V [cm3]	Dmin	Dmax	Dmedian	Dmean	Dstd
CTV	343.5	3342.0	3933.8	3623.5	3632.6	84.5
lung	2331.0	42.3	3863.9	1500.7	1826.0	1078.9
esophagus	30.5	121.4	3705.2	1870.7	1990.7	1412.3
spinal cord	29.8	44.5	3599.2	1484.6	1464.0	1249.6
heart	581.7	220.4	3654.9	1367.3	1534.3	912.2
PTV	779.8	2782.2	3963.1	3603.7	3600.0	136.9

Case 17

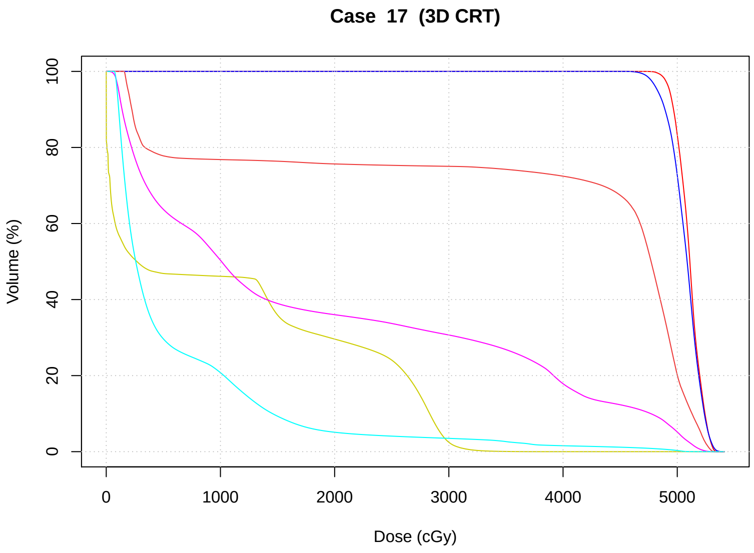

Structure	V [cm3]	Dmin	Dmax	Dmedian	Dmean	Dstd
CTV	246.8	4721.5	5362.6	5110.2	5105.3	102.9
PTV	696.9	4434.9	5413.2	5086.4	5072.7	133.1
spinal cord	70.0	0.0	3798.4	268.8	1088.2	1180.6
lung	3709.6	15.7	5337.8	1009.2	1814.5	1760.9
esophagus	21.5	156.3	5323.8	4770.8	3713.4	1957.0
heart	643.8	69.2	5187.0	258.5	606.7	837.3

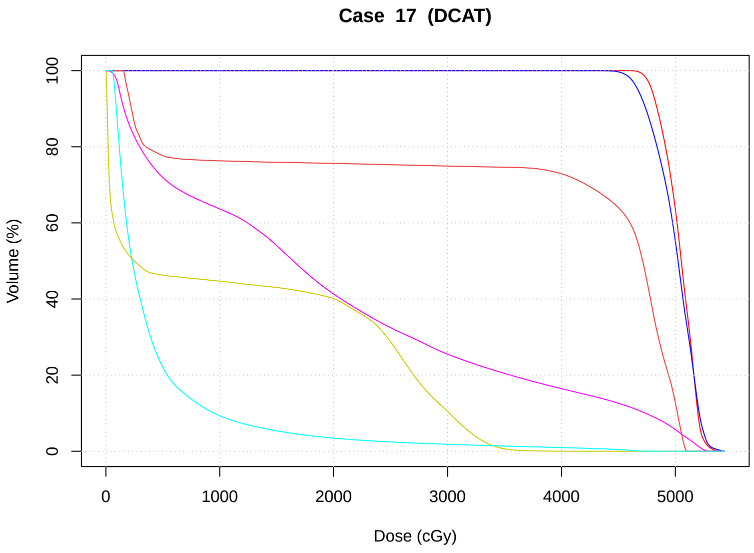

Structure	V [cm3]	Dmin	Dmax	Dmedian	Dmean	Dstd
CTV	246.8	4608.4	5382.2	5052.3	5037.8	136.2
PTV	696.9	4291.9	5431.2	5023.8	5000.0	178.8
spinal cord	70.0	3.0	4075.7	251.5	1216.2	1306.6
lung	3709.6	16.8	5369.6	1644.7	1957.4	1619.9
esophagus	21.5	151.4	5109.1	4713.3	3648.7	1926.2
heart	643.8	55.1	4757.2	230.3	445.1	646.6

Case 18

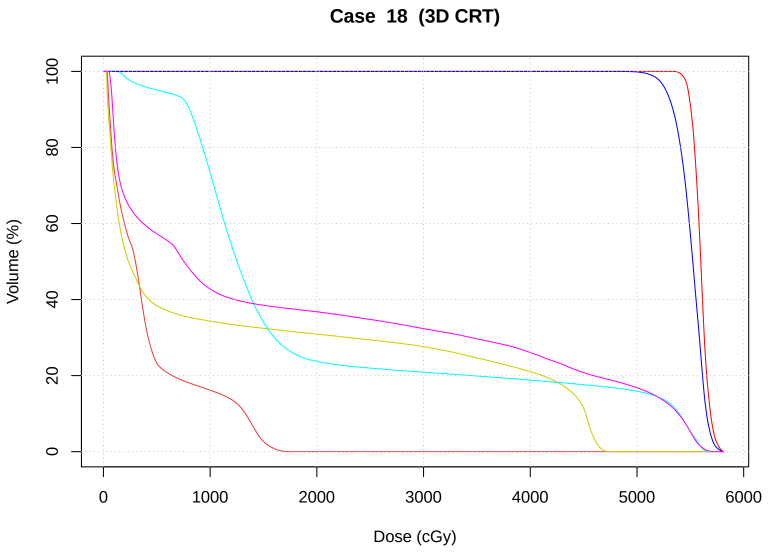

Structure	V [cm3]	Dmin	Dmax	Dmedian	Dmean	Dstd
CTV	256.8	5331.5	5815.3	5598.0	5597.5	71.7
esophagus	21.7	34.2	1755.1	299.7	433.4	448.6
spinal cord	44.4	25.6	4740.0	235.0	1420.8	1826.2
heart	714.8	121.5	5730.8	1251.5	1989.5	1674.0
PTV	595.2	4800.8	5815.3	5523.0	5507.3	127.8
lung	2890.3	44.1	5746.7	755.1	1914.0	2097.0

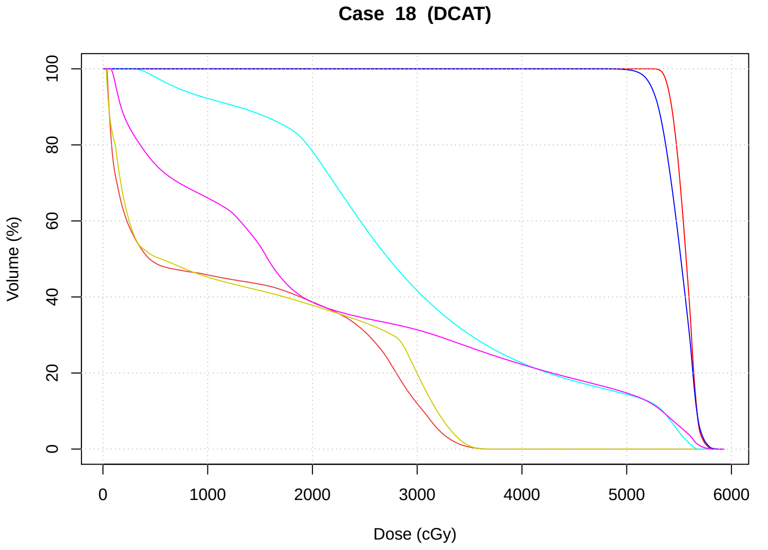

Structure	V [cm3]	Dmin	Dmax	Dmedian	Dmean	Dstd
CTV	256.8	5237.7	5824.5	5567.6	5559.1	93.7
esophagus	21.7	35.4	3743.4	445.1	1296.4	1275.8
spinal cord	44.4	30.7	3676.0	553.5	1351.9	1330.2
heart	714.8	281.0	5691.4	2729.1	2980.9	1379.8
PTV	595.2	4752.0	5928.6	5517.0	5500.0	147.2
lung	2890.3	61.1	5883.8	1573.2	2172.5	1852.7

Case 19

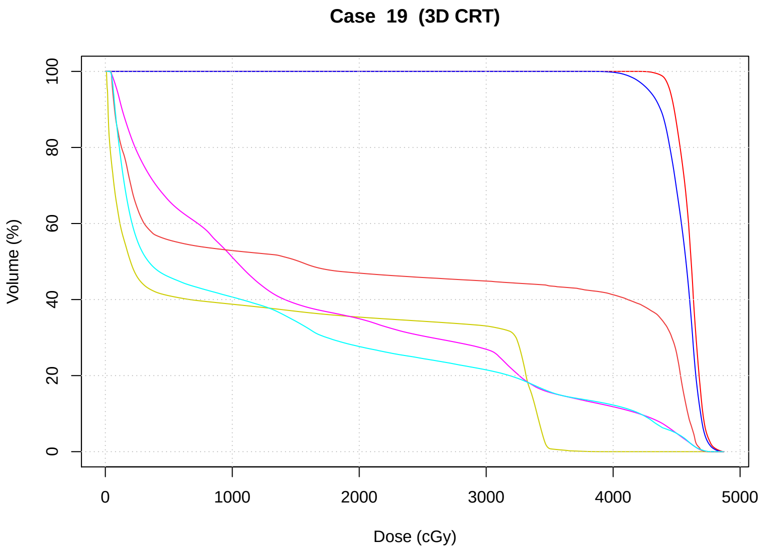

Structure	V [cm3]	Dmin	Dmax	Dmedian	Dmean	Dstd
CTV	148.8	4204.8	4872.9	4614.4	4603.9	89.1
PTV	403.7	3760.2	4872.9	4573.5	4545.4	135.7
spinal cord	72.1	6.5	3964.0	198.3	1289.9	1512.6
lung	4500.7	26.1	4819.1	1031.3	1633.5	1506.7
esophagus	31.4	44.2	4737.9	1529.2	2219.8	2048.6
heart	711.3	36.3	4795.3	340.3	1334.8	1564.0

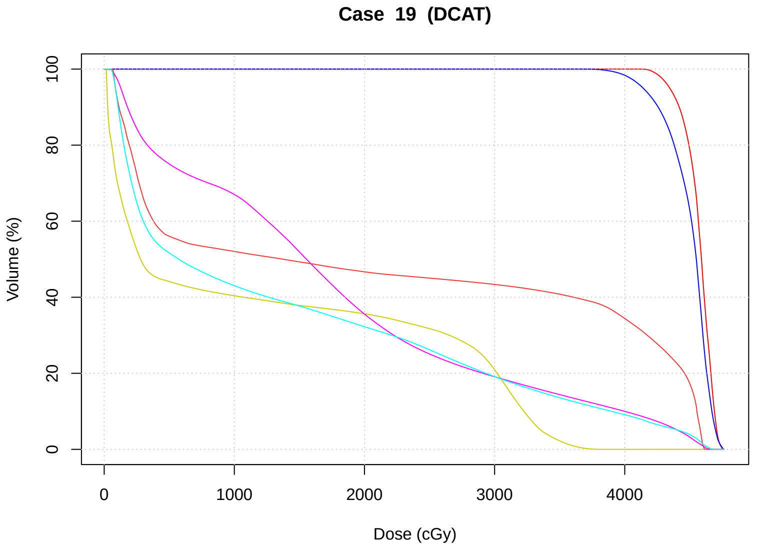

Structure	V [cm3]	Dmin	Dmax	Dmedian	Dmean	Dstd
CTV	148.8	4091.8	4761.8	4589.8	4569.9	108.7
PTV	403.7	3676.9	4762.5	4550.0	4500.0	170.3
spinal cord	72.1	15.1	3831.0	279.4	1240.9	1370.9
lung	4500.7	40.5	4724.6	1554.4	1744.3	1329.3
esophagus	31.4	62.3	4628.8	1374.2	2137.1	1941.6
heart	711.3	50.9	4681.9	573.1	1398.7	1479.1

Case 20

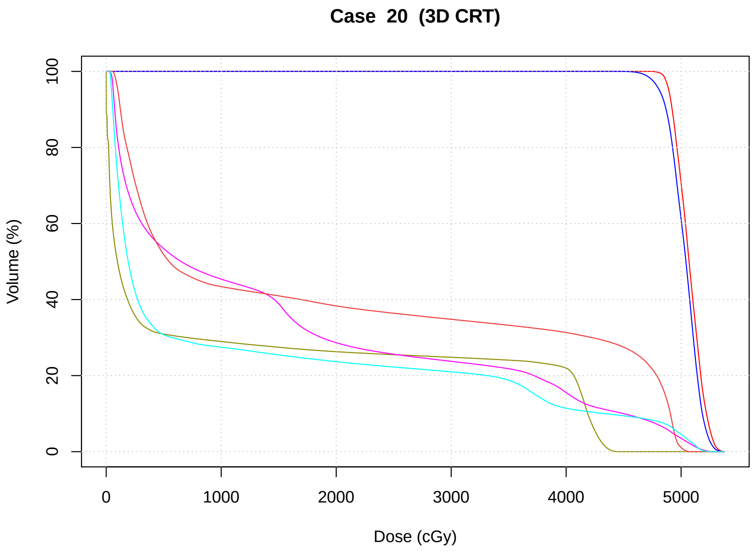

Structure	V [cm3]	Dmin	Dmax	Dmedian	Dmean	Dstd
CTV	227.7	4741.6	5376.8	5068.9	5070.0	112.2
PTV	576.9	4415.9	5376.8	5041.7	5031.0	126.9
spinal cord PORV	193.5	0.0	4456.1	97.3	1167.7	1745.6
lung	4808.8	12.4	5339.9	651.8	1534.7	1702.6
esophagus	88.4	56.7	5072.7	546.7	1949.8	2048.8
heart	610.7	30.4	5266.3	191.1	1151.2	1709.1

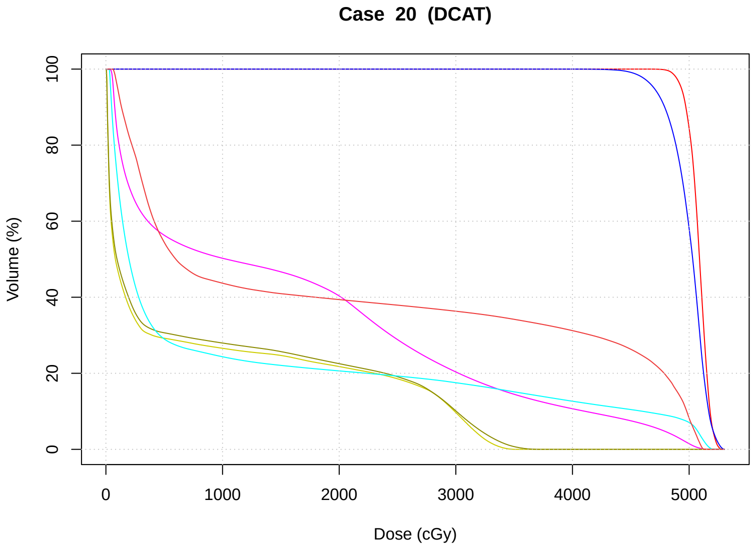

Structure	V [cm3]	Dmin	Dmax	Dmedian	Dmean	Dstd
lung	4808.8	27.5	5195.0	1034.2	1593.5	1572.5
CTV	227.7	4669.0	5283.6	5089.1	5082.1	82.3
spinal cord	63.9	3.7	3515.3	81.1	778.3	1176.9
spinal cord PORV	193.5	2.8	3733.5	95.3	815.9	1198.0
heart	610.7	28.1	5218.6	197.9	1063.5	1679.8
PTV	576.9	3992.8	5302.8	5030.7	5000.0	156.0
esophagus	88.4	57.8	5136.2	599.4	2004.8	2053.6

Case 21

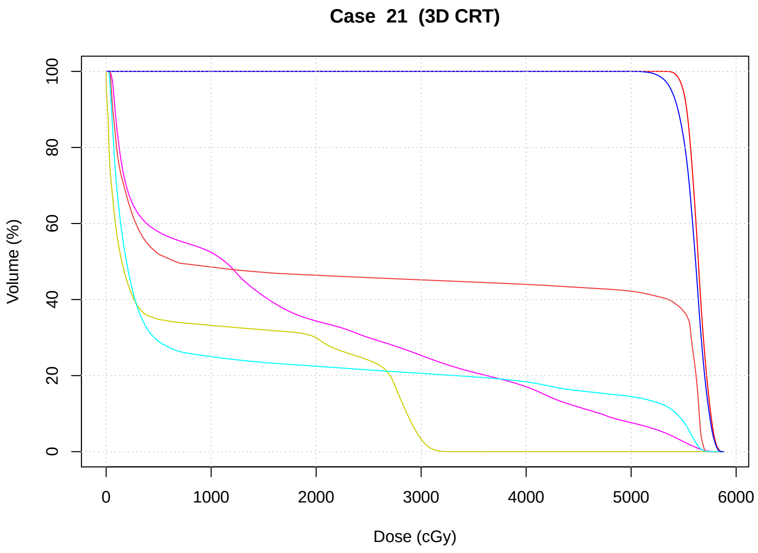

Structure	V [cm3]	Dmin	Dmax	Dmedian	Dmean	Dstd
CTV	245.5	5305.4	5883.9	5639.8	5640.3	87.4
lung	3276.1	26.5	5851.2	1129.8	1711.7	1784.6
esophagus	31.5	35.3	5737.1	658.9	2640.1	2619.3
spinal cord	45.9	0.0	3348.4	149.2	939.5	1207.6
heart	539.9	23.6	5775.8	193.2	1263.6	2004.1
PTV	429.2	4967.9	5883.9	5613.6	5601.1	118.6

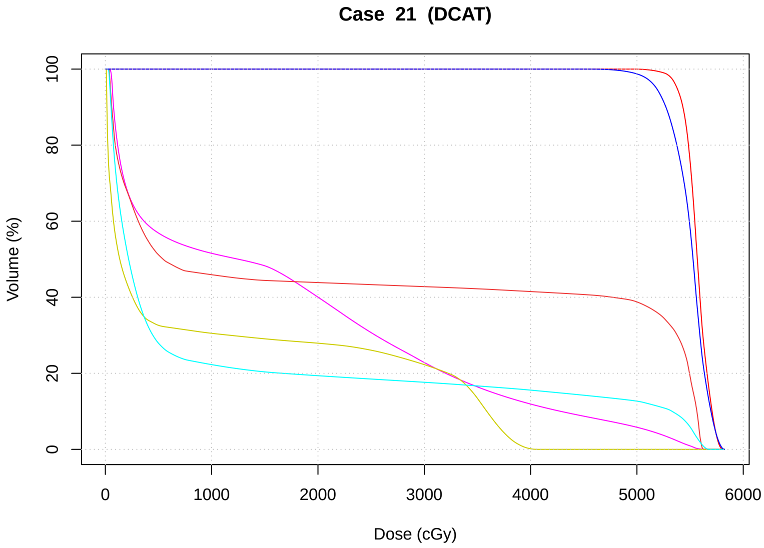

Structure	V [cm3]	Dmin	Dmax	Dmedian	Dmean	Dstd
CTV	245.5	4961.9	5814.8	5568.0	5565.4	109.5
lung	3276.1	34.6	5707.6	1251.2	1678.6	1668.7
esophagus	31.5	34.6	5626.8	543.5	2461.7	2530.3
spinal cord	45.9	8.5	4076.4	135.6	1057.0	1472.1
heart	539.9	29.0	5686.7	219.0	1139.3	1882.5
PTV	429.2	4495.6	5823.1	5527.8	5500.0	170.1

Case 22

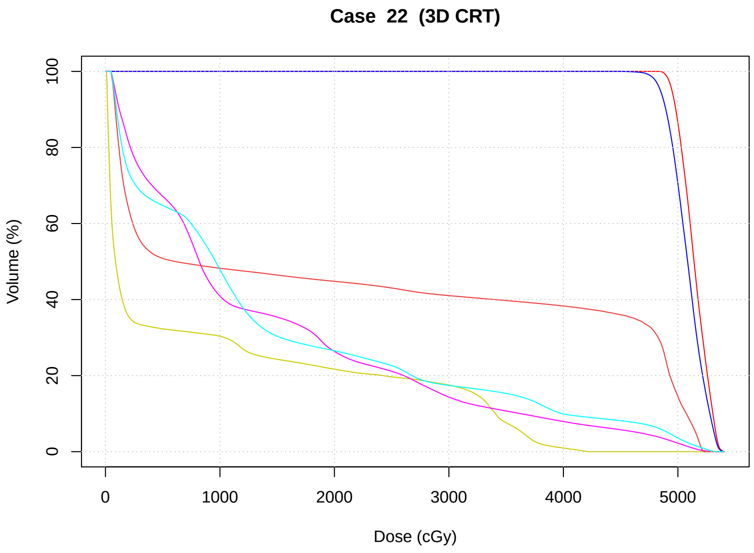

Structure	V [cm3]	Dmin	Dmax	Dmedian	Dmean	Dstd
CTV	152.3	4831.4	5406.8	5141.2	5142.1	118.9
PTV	295.4	4444.0	5406.8	5085.1	5083.0	143.8
spinal cord	45.0	6.8	4225.7	88.0	898.9	1342.7
lung	4509.9	34.2	5380.6	819.9	1395.1	1389.1
esophagus	42.5	46.0	5232.5	611.3	2225.1	2244.0
heart	843.6	42.1	5350.9	961.7	1450.5	1514.3

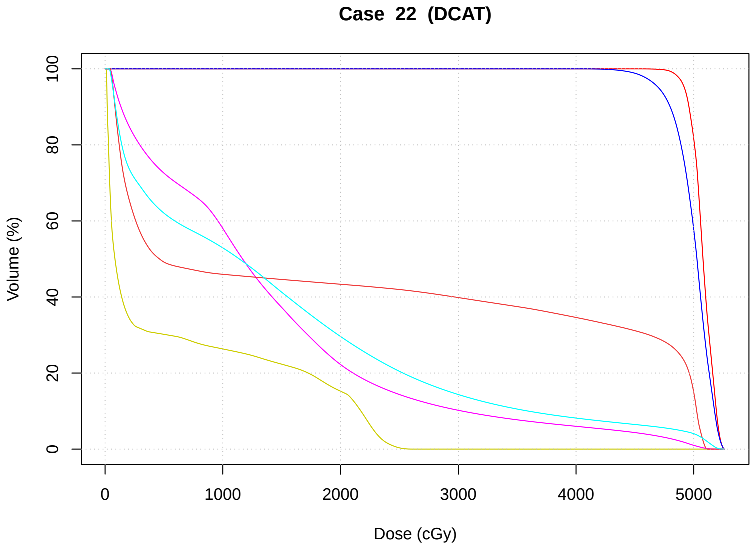

Structure	V [cm3]	Dmin	Dmax	Dmedian	Dmean	Dstd
CTV	152.3	4539.0	5258.6	5078.3	5076.3	90.9
PTV	295.4	4051.2	5258.5	5027.0	5000.0	157.4
spinal cord	45.0	11.4	2607.7	84.0	597.5	852.2
lung	4509.9	28.6	5214.3	1168.0	1403.0	1200.4
esophagus	42.5	44.0	5117.1	462.5	2116.9	2187.9
heart	843.6	34.5	5255.3	1140.5	1463.4	1428.1

Case 23

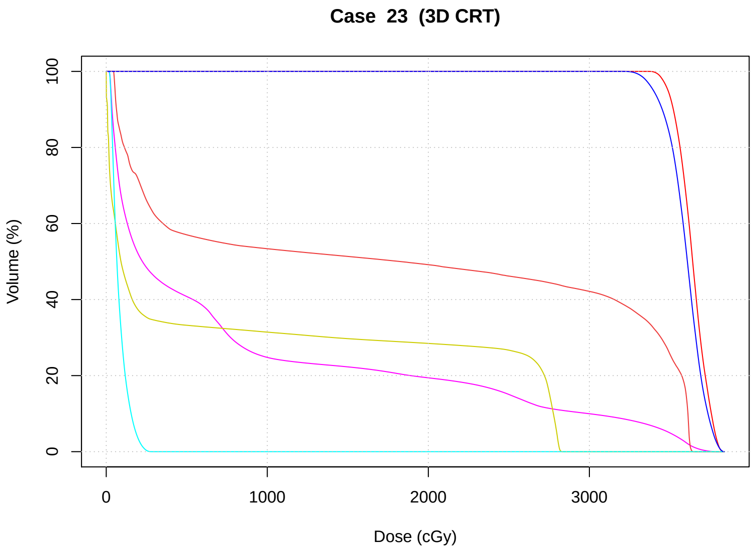

Structure	V [cm3]	Dmin	Dmax	Dmedian	Dmean	Dstd
CTV	151.4	3369.0	3838.7	3641.3	3639.2	87.8
lung	3368.1	10.9	3811.2	224.6	850.6	1139.1
esophagus	17.4	44.8	3649.0	1825.3	1809.0	1572.9
spinal cord	63.9	0.0	2842.6	91.5	873.4	1194.0
heart	313.5	18.9	288.7	66.0	81.0	49.2
PTV	319.3	3203.3	3838.7	3608.5	3600.0	108.4

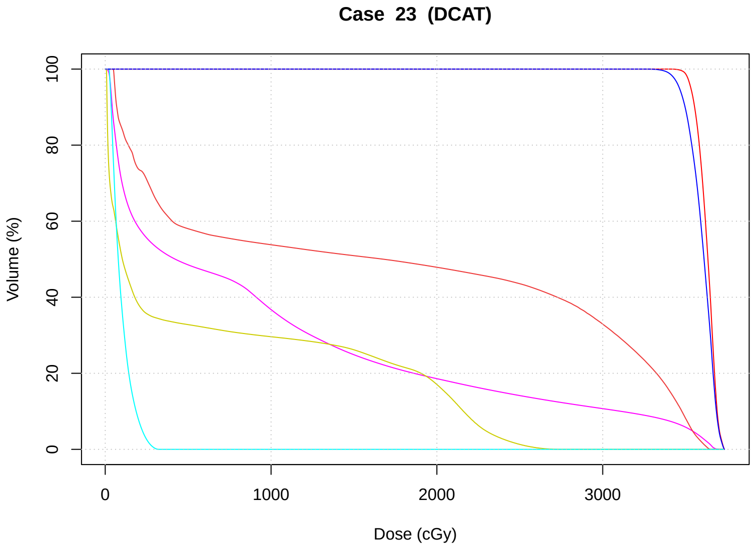

Structure	V [cm3]	Dmin	Dmax	Dmedian	Dmean	Dstd
CTV	151.4	3402.8	3734.1	3634.5	3627.9	52.6
lung	3368.1	7.2	3713.2	422.5	977.2	1129.8
esophagus	17.4	48.8	3651.4	1678.0	1696.1	1443.0
spinal cord	63.9	6.1	2749.0	103.3	659.8	889.2
heart	313.5	23.0	330.9	78.6	96.4	60.1
PTV	319.3	3246.1	3734.1	3611.1	3600.0	74.1

Case 24

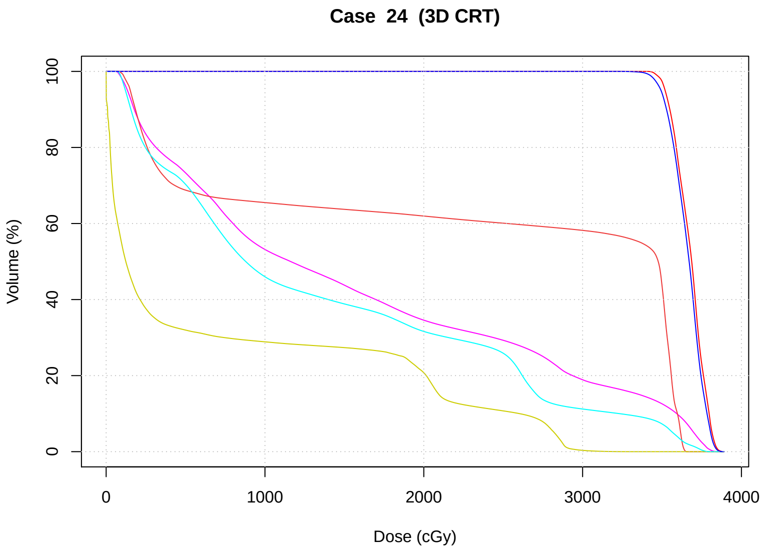

Structure	V [cm3]	Dmin	Dmax	Dmedian	Dmean	Dstd
CTV	314.1	3407.0	3890.1	3687.0	3676.4	91.6
lung	3866.4	47.3	3881.9	1161.6	1564.1	1247.3
esophagus	31.9	77.0	3653.9	3476.0	2295.1	1523.2
spinal cord	55.4	0.1	3292.8	123.8	738.2	1016.9
heart	824.4	69.2	3802.7	878.9	1372.9	1152.6
PTV	726.6	3211.1	3890.1	3670.9	3660.3	96.4

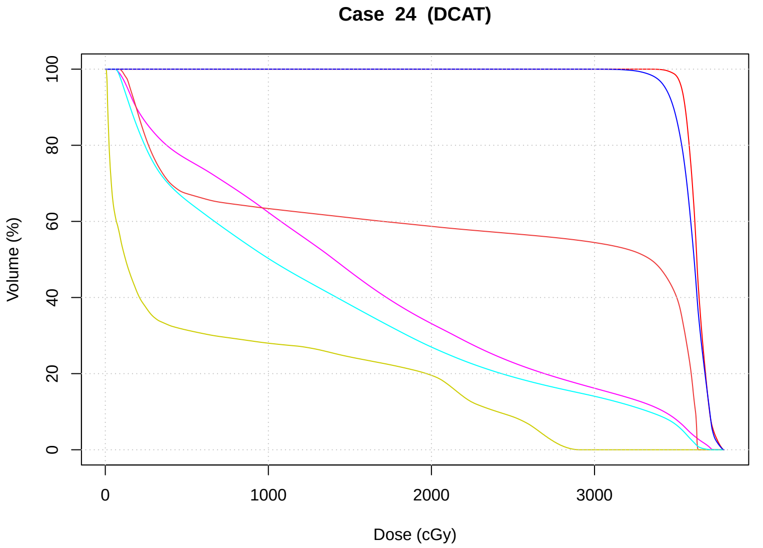

Structure	V [cm3]	Dmin	Dmax	Dmedian	Dmean	Dstd
CTV	314.1	3326.0	3793.7	3627.4	3629.4	60.0
lung	3866.4	43.7	3758.2	1403.1	1565.9	1122.3
esophagus	31.9	87.6	3638.8	3343.4	2207.5	1529.9
spinal cord	55.4	5.3	2906.7	124.1	693.3	961.1
heart	824.4	59.9	3726.9	1009.8	1335.3	1135.3
PTV	726.6	2996.6	3793.9	3611.0	3600.0	91.7

Case 25

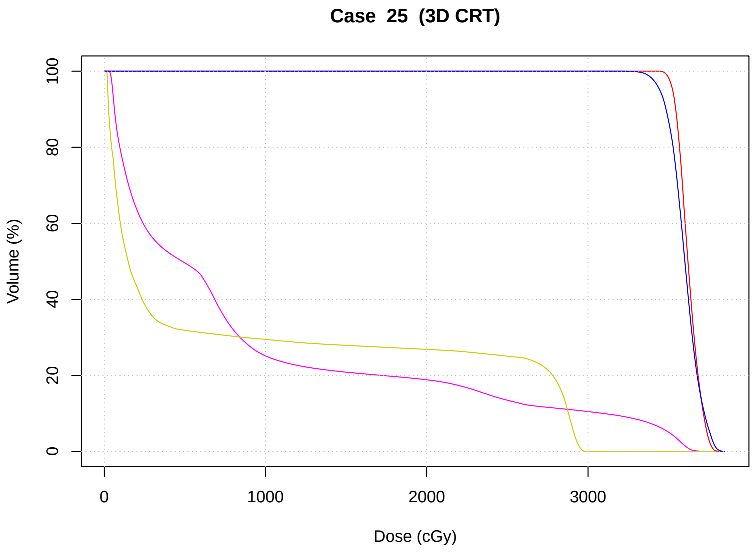

Structure	V [cm3]	Dmin	Dmax	Dmedian	Dmean	Dstd
lung	3311.3	24.0	3772.9	483.5	909.4	1093.9
spinal cord	39.3	13.5	2988.3	148.1	883.9	1191.7
CTV	341.0	3436.5	3822.3	3619.5	3624.3	64.8
PTV	763.9	3197.7	3844.5	3600.3	3600.1	91.7

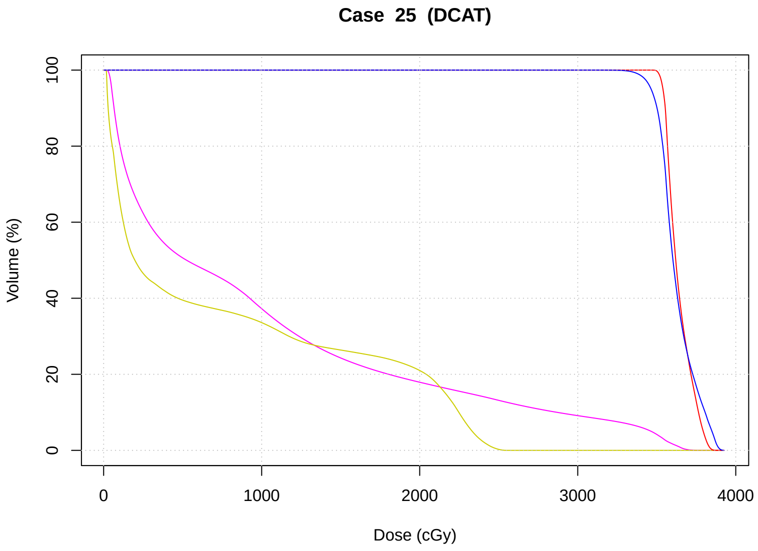

Structure	V [cm3]	Dmin	Dmax	Dmedian	Dmean	Dstd
lung	3311.3	18.6	3763.6	524.9	986.9	1069.5
spinal cord	39.3	16.0	2547.7	197.6	770.9	891.5
CTV	341.0	3462.0	3873.2	3620.2	3639.9	80.1
PTV	763.9	3085.3	3925.4	3602.7	3626.2	114.7

Case 26

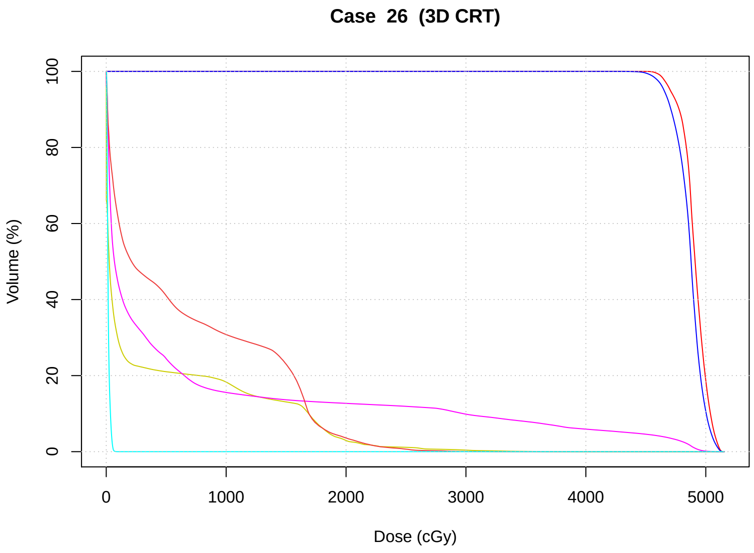

Structure	V [cm3]	Dmin	Dmax	Dmedian	Dmean	Dstd
CTV	98.7	4484.2	5155.7	4910.9	4908.2	103.9
PTV	186.6	4230.6	5155.7	4877.6	4866.1	114.8
spinal cord	65.7	0.0	4020.5	25.5	362.6	678.3
lung	5582.0	0.0	5155.7	68.4	657.7	1293.2
esophagus	48.1	0.0	3372.1	211.0	641.6	725.0
heart	500.4	0.5	101.3	13.7	17.1	12.0

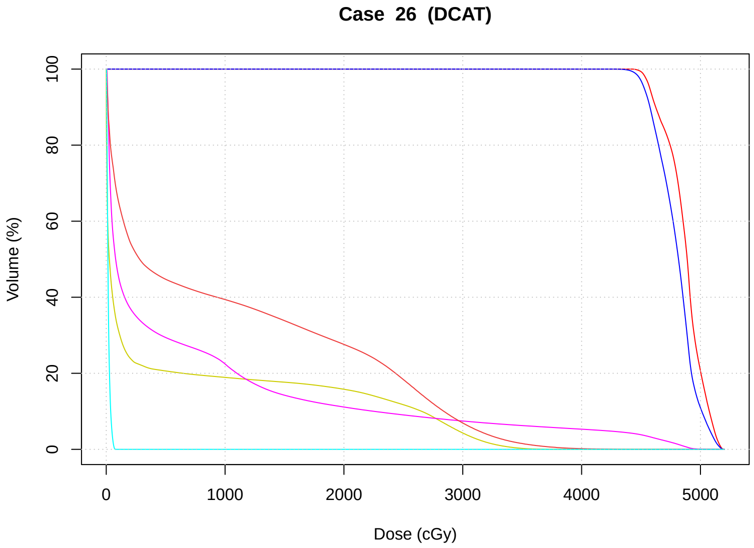

Structure	V [cm3]	Dmin	Dmax	Dmedian	Dmean	Dstd
CTV	98.7	4420.5	5201.9	4888.5	4870.2	157.1
PTV	186.6	4263.7	5201.9	4815.3	4800.0	162.6
spinal cord	65.7	0.0	3809.9	26.3	531.7	1018.5
lung	5582.0	1.9	5135.2	79.2	672.9	1197.4
esophagus	48.1	2.8	4436.6	281.1	1030.7	1170.9
heart	500.4	2.0	89.4	14.1	18.0	13.6

Case 27

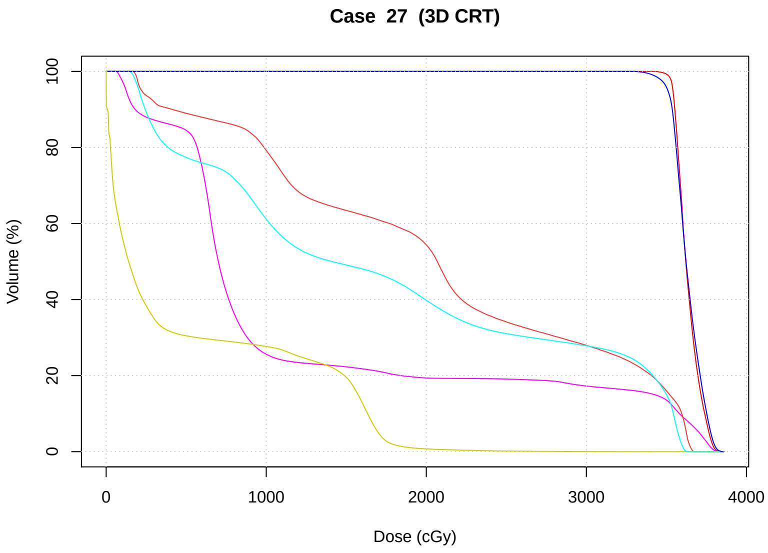

Structure	V [cm3]	Dmin	Dmax	Dmedian	Dmean	Dstd
CTV	1135.6	3413.4	3850.0	3621.0	3633.5	70.0
lung	2399.6	56.8	3850.0	700.7	1208.8	1168.7
esophagus	32.4	165.2	3671.8	2068.4	2039.8	1115.7
spinal cord	77.9	0.0	3274.4	140.3	522.6	667.9
heart	871.0	139.3	3647.8	1413.0	1749.2	1246.0
PTV	1739.6	3155.6	3859.7	3622.6	3631.8	85.0

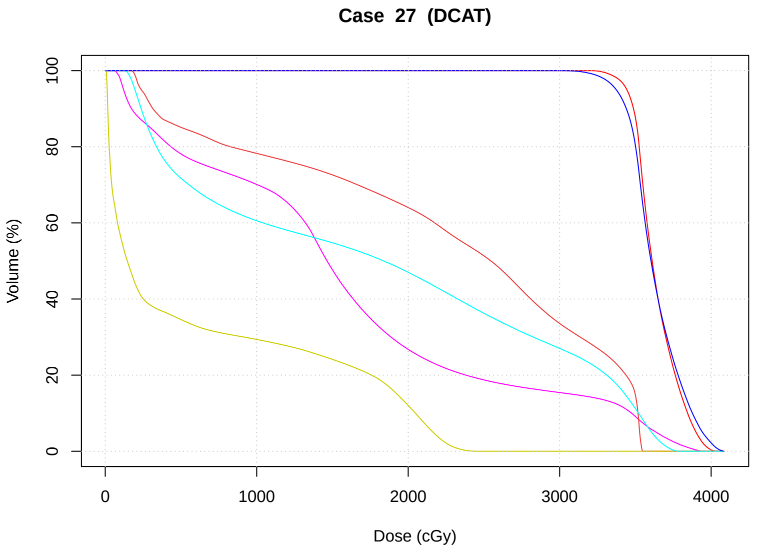

Structure	V [cm3]	Dmin	Dmax	Dmedian	Dmean	Dstd
CTV	1135.6	3186.5	4060.0	3610.8	3637.2	140.2
lung	2399.6	50.5	3969.0	1464.6	1575.8	1077.9
esophagus	32.4	172.4	3550.9	2546.3	2232.9	1147.0
spinal cord	77.9	6.1	2454.4	145.7	643.5	817.0
heart	871.0	122.5	3791.3	1838.5	1792.9	1280.5
PTV	1739.6	2972.9	4084.7	3603.0	3630.0	171.4

Case 28

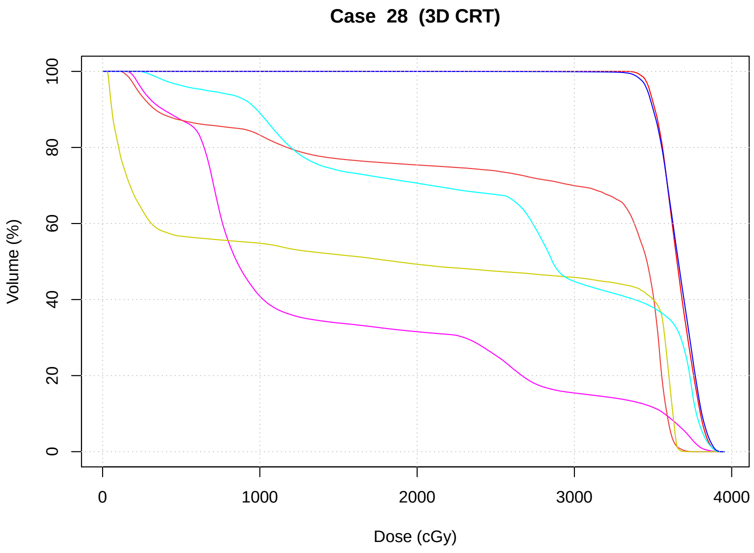

Structure	V [cm3]	Dmin	Dmax	Dmedian	Dmean	Dstd
CTV	1928.2	3319.4	3937.7	3652.4	3655.5	108.1
lung	5341.6	109.3	3917.6	853.4	1473.9	1150.8
esophagus	63.2	111.4	3763.1	3461.5	2752.0	1230.7
spinal cord	82.9	23.2	3715.0	1861.6	1877.1	1635.9
heart	579.0	231.6	3954.1	2860.1	2651.0	1128.2
PTV	2686.5	237.2	3943.0	3661.1	3658.1	123.0

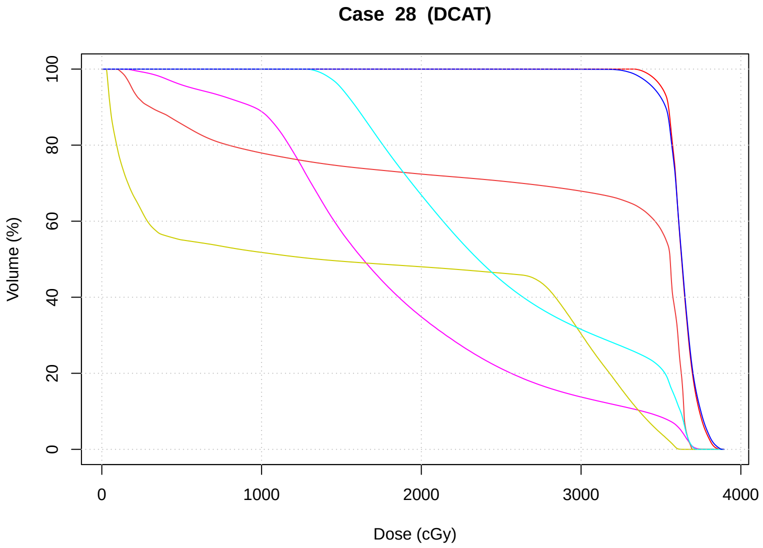

Structure	V [cm3]	Dmin	Dmax	Dmedian	Dmean	Dstd
CTV	1928.2	3329.1	3885.4	3629.2	3633.9	80.9
lung	5341.6	105.6	3806.5	1634.7	1843.9	868.1
esophagus	63.2	91.4	3699.0	3557.3	2705.6	1336.8
spinal cord	82.9	27.2	3635.5	1354.1	1630.3	1453.6
heart	579.0	1269.0	3721.5	2354.8	2520.0	761.4
PTV	2686.5	694.7	3894.4	3630.8	3629.3	100.8

Case 29

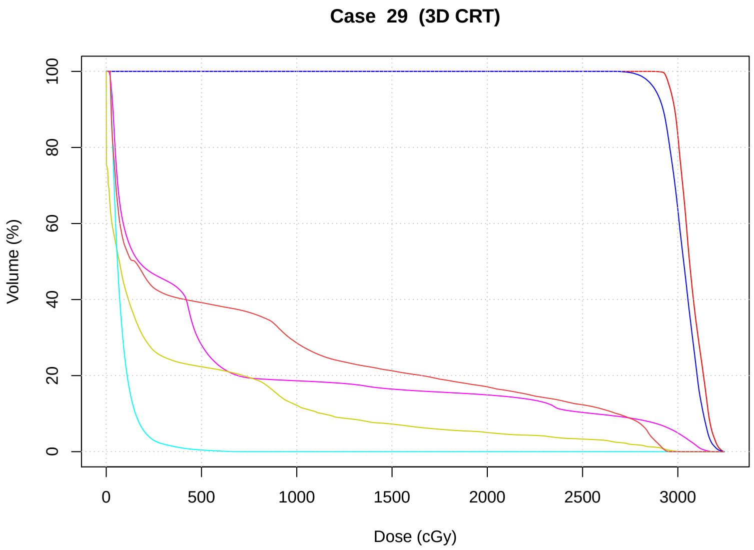

Structure	V [cm3]	Dmin	Dmax	Dmedian	Dmean	Dstd
CTV	173.8	2859.0	3244.7	3061.2	3067.8	67.8
lung	3627.8	9.3	3218.3	170.4	637.4	943.6
spinal cord	59.9	0.0	3021.9	68.9	364.0	655.0
heart	756.6	16.1	743.0	58.5	80.3	70.7
PTV	430.1	2653.1	3244.7	3030.4	3027.3	84.7
esophagus	37.2	19.4	2955.1	150.6	751.4	978.2

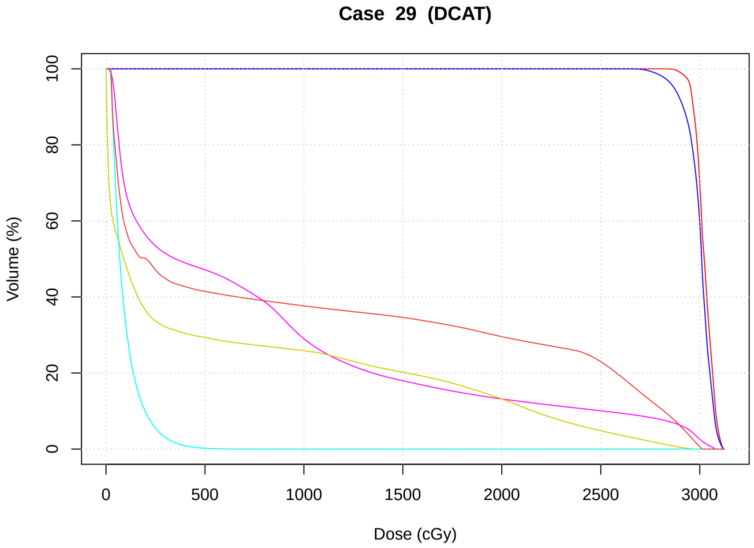

Structure	V [cm3]	Dmin	Dmax	Dmedian	Dmean	Dstd
CTV	173.8	2836.2	3124.9	3022.8	3023.4	44.3
lung	3627.8	9.6	3101.5	352.2	784.2	908.1
spinal cord	59.9	1.2	2977.0	90.5	588.6	876.9
heart	756.6	16.4	722.0	69.5	96.0	78.2
PTV	430.1	2637.6	3124.9	3009.9	3000.0	65.6
esophagus	37.2	24.0	3014.4	203.4	1001.9	1166.3

Case 30

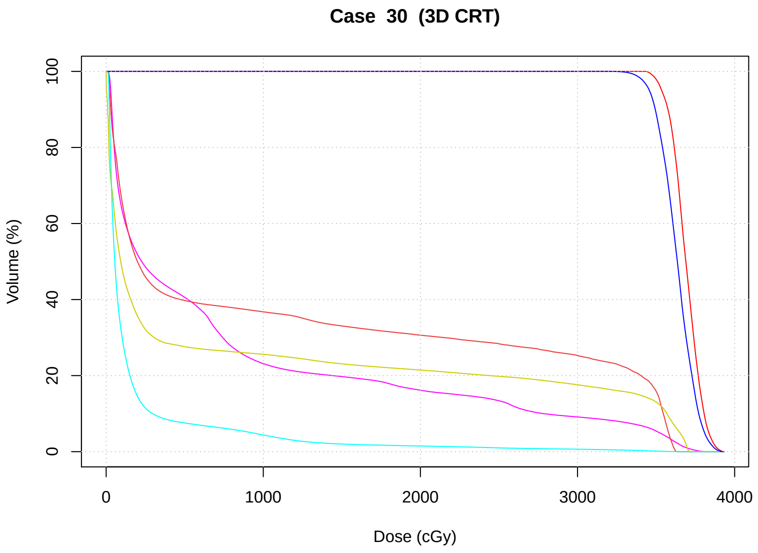

Structure	V [cm3]	Dmin	Dmax	Dmedian	Dmean	Dstd
CTV	195.4	3428.5	3932.1	3689.5	3690.0	89.4
lung	6536.0	0.0	3882.3	225.7	797.7	1098.4
esophagus	33.7	15.9	3636.9	199.2	1196.1	1465.8
spinal cord	48.4	0.0	3718.1	92.8	854.9	1359.1
heart	790.1	11.5	3679.3	54.8	176.1	415.4
PTV	499.3	3168.1	3932.1	3635.3	3633.4	107.8

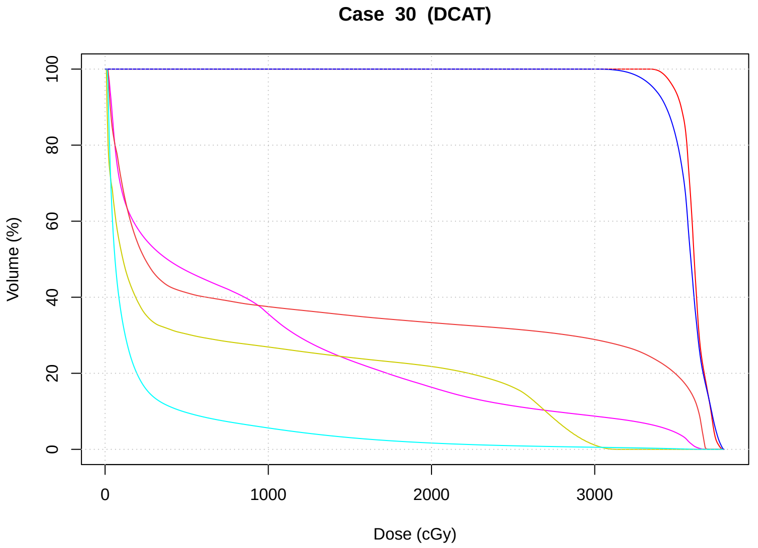

Structure	V [cm3]	Dmin	Dmax	Dmedian	Dmean	Dstd
CTV	195.4	3336.0	3784.8	3609.6	3612.0	70.9
lung	6536.0	6.6	3721.6	380.9	909.8	1067.5
esophagus	33.7	17.2	3703.2	245.0	1297.2	1530.7
spinal cord	48.4	7.7	3140.0	109.3	736.9	1067.7
heart	790.1	11.2	3649.0	60.8	209.8	448.1
PTV	499.3	3009.7	3791.9	3588.8	3578.5	112.1

Case 31

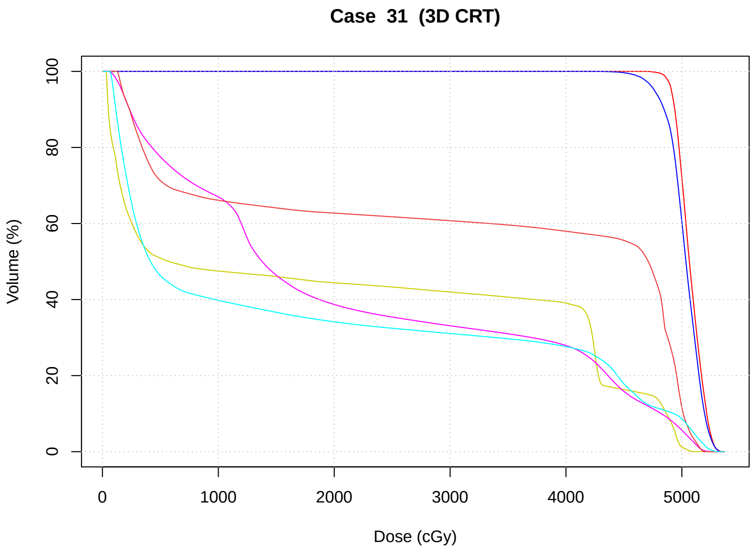

Structure	V [cm3]	Dmin	Dmax	Dmedian	Dmean	Dstd
CTV	342.6	4678.1	5367.4	5064.6	5070.5	105.4
PTV	837.4	4190.8	5367.4	5034.8	5026.3	142.7
spinal cord	43.8	32.7	5103.8	575.6	2068.3	2079.2
lung	3692.0	51.5	5322.9	1378.1	2137.4	1751.9
esophagus	37.9	127.4	5194.0	4710.1	3135.4	2126.6
heart	733.1	60.8	5316.9	414.1	1744.9	1981.6

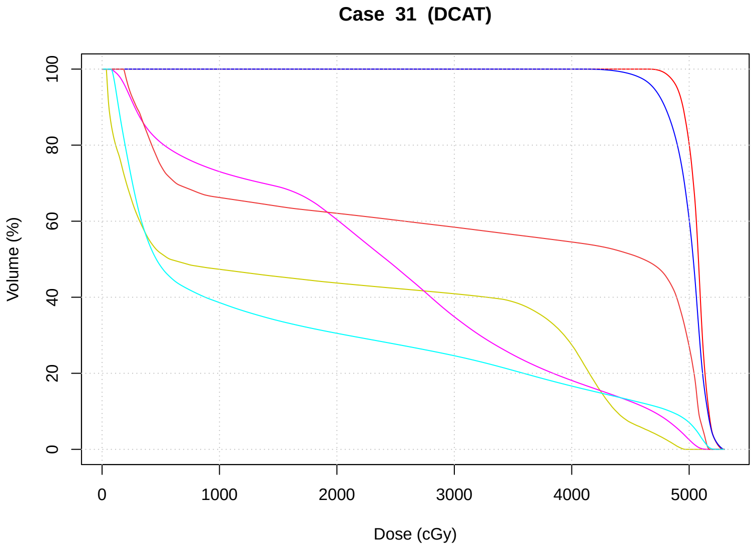

Structure	V [cm3]	Dmin	Dmax	Dmedian	Dmean	Dstd
CTV	342.6	4643.6	5293.4	5078.9	5066.2	89.5
PTV	837.4	4098.0	5300.7	5035.1	5000.0	154.8
spinal cord	43.8	35.5	4968.6	579.8	1936.9	1909.3
lung	3692.0	61.5	5194.4	2418.2	2373.8	1529.7
esophagus	37.9	182.4	5176.9	4610.3	3118.7	2109.2
heart	733.1	77.0	5214.6	462.0	1538.8	1766.4

Case 32

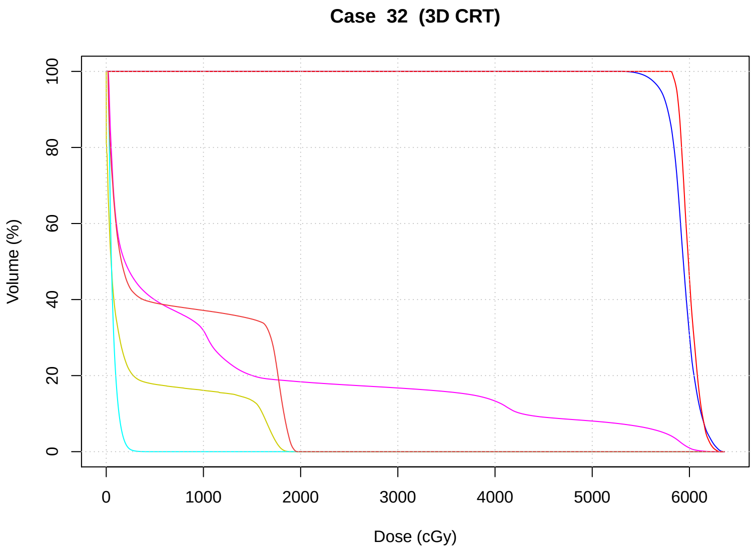

Structure	V [cm3]	Dmin	Dmax	Dmedian	Dmean	Dstd
spinal cord	61.4	0.0	1906.4	50.3	322.9	579.5
heart	520.6	17.1	451.9	53.1	68.2	46.1
PTV	190.5	5280.5	6360.2	5937.2	5940.0	142.8
lung	4004.7	12.6	6293.1	191.8	1151.3	1774.4
CTV	50.1	5803.2	6317.2	5989.8	6001.2	92.9
esophagus	32.3	17.3	1970.8	157.0	721.9	802.2

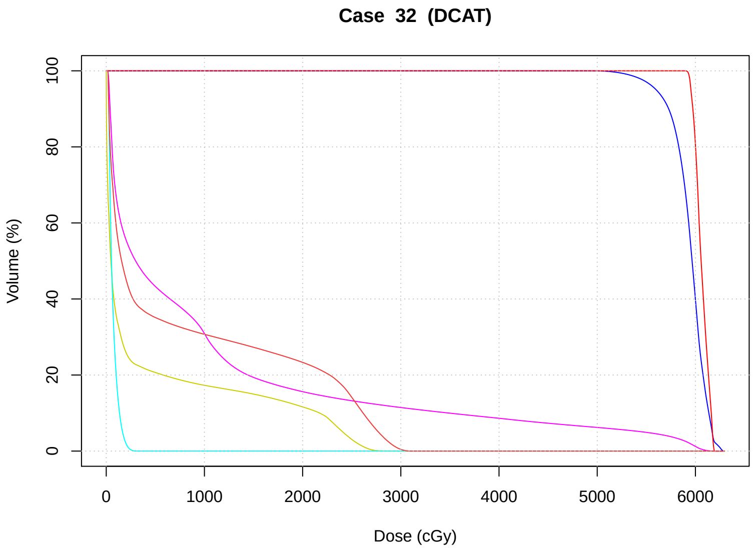

Structure	V [cm3]	Dmin	Dmax	Dmedian	Dmean	Dstd
spinal cord	61.4	0.0	2873.4	47.5	436.6	799.7
heart	520.6	15.5	334.6	53.7	69.1	46.2
PTV	190.5	4895.3	6295.6	5966.0	5940.0	177.3
lung	4004.7	9.2	6188.2	299.3	1037.8	1561.6
CTV	50.1	5897.4	6197.3	6058.0	6063.3	67.7
esophagus	32.3	17.4	3086.5	156.6	810.9	1048.7

Case 33

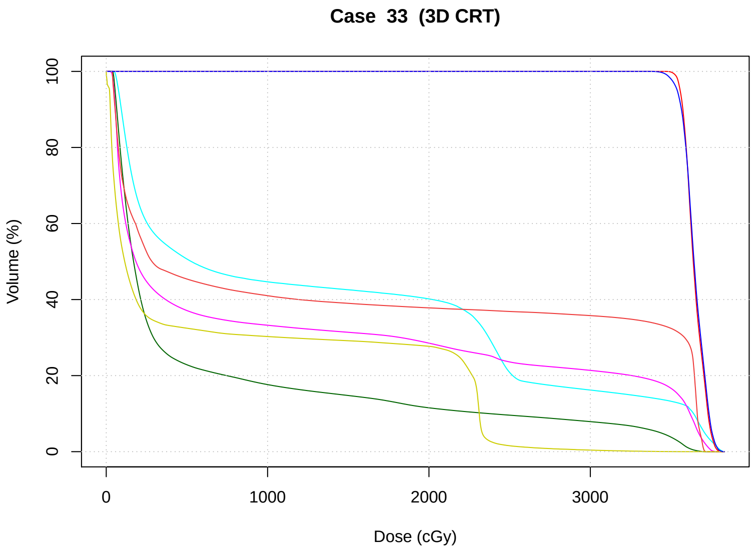

Structure	V [cm3]	Dmin	Dmax	Dmedian	Dmean	Dstd
liver	816.6	38.3	3724.1	169.4	612.8	995.5
heart	355.0	45.3	3830.9	527.6	1357.5	1350.2
CTV	404.7	3466.1	3826.4	3637.9	3645.6	59.2
lung	2515.4	19.1	3806.0	184.4	1118.0	1432.8
esophagus	15.0	35.9	3719.9	283.6	1495.2	1638.6
spinal cord	64.4	0.0	3582.7	114.5	754.2	1001.4
PTV	636.3	3353.4	3831.2	3642.7	3646.4	67.2

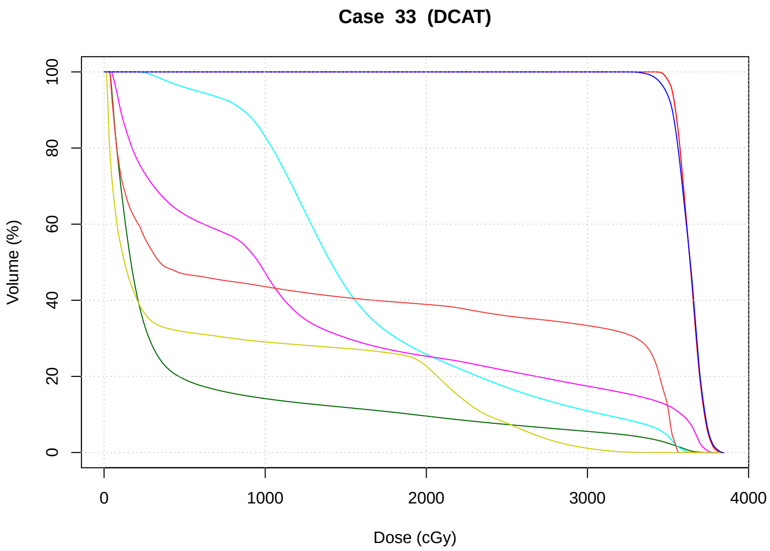

Structure	V [cm3]	Dmin	Dmax	Dmedian	Dmean	Dstd
liver	816.6	33.1	3761.7	166.5	522.5	890.0
heart	355.0	210.1	3657.3	1406.5	1651.6	821.6
CTV	404.7	3409.7	3847.2	3636.4	3635.8	69.0
lung	2515.4	38.5	3788.3	958.7	1297.5	1233.0
esophagus	15.0	35.2	3568.4	345.8	1454.2	1524.2
spinal cord	64.4	12.9	3390.9	126.8	737.0	998.6
PTV	636.3	3213.3	3847.2	3638.0	3630.0	83.2

Case 34

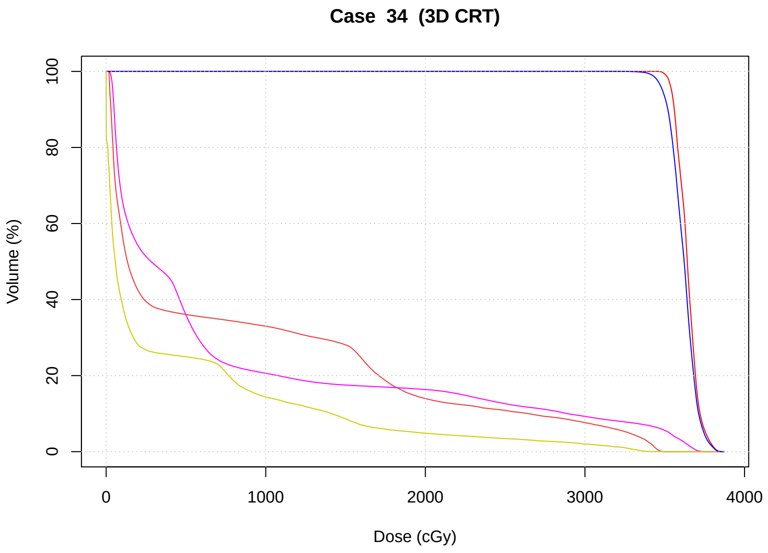

Structure	V [cm3]	Dmin	Dmax	Dmedian	Dmean	Dstd
CTV	299.9	3444.3	3871.2	3641.4	3641.7	64.6
lung	4838.4	11.9	3802.1	280.9	761.8	1083.8
esophagus	43.3	15.7	3490.8	133.1	795.7	1059.3
spinal cord	72.0	0.0	3421.7	56.9	396.9	711.2
PTV	613.4	3184.6	3871.2	3621.7	3617.4	78.8

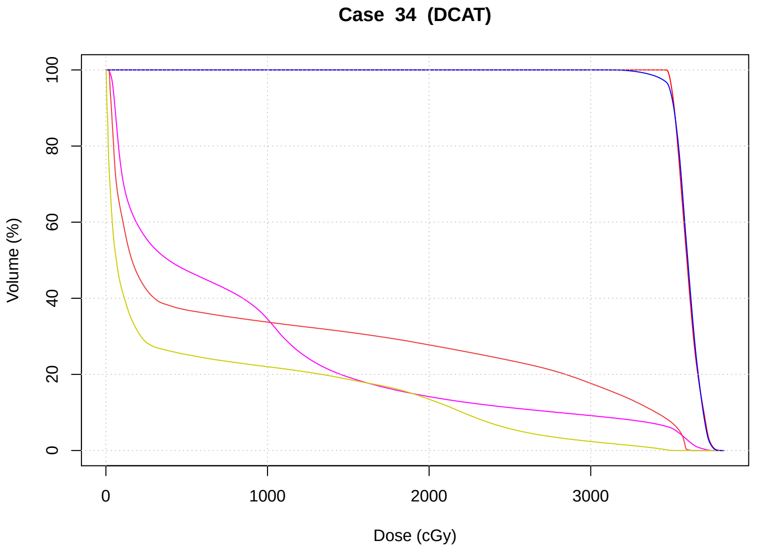

Structure	V [cm3]	Dmin	Dmax	Dmedian	Dmean	Dstd
CTV	299.9	3452.9	3824.5	3595.3	3601.3	67.3
lung	4838.4	6.9	3797.1	389.1	886.8	1053.5
esophagus	43.3	19.2	3635.9	161.4	1041.4	1344.9
spinal cord	72.0	1.7	3528.4	64.7	551.4	913.4
PTV	613.4	3103.3	3824.5	3600.8	3600.0	76.6

Case 35

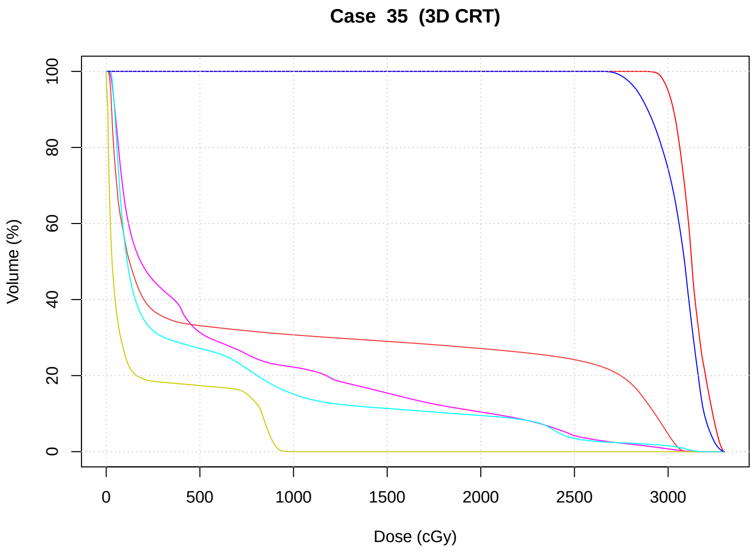

Structure	V [cm3]	Dmin	Dmax	Dmedian	Dmean	Dstd
CTV	29.3	2878.1	3300.8	3125.8	3128.4	77.3
lung	3053.9	7.7	3215.7	184.4	599.3	792.5
esophagus	35.9	11.6	3103.1	123.8	897.9	1209.3
spinal cord	73.5	0.0	1020.1	31.2	178.4	302.7
heart	749.8	23.0	3207.2	112.0	487.9	759.2
PTV	145.2	2628.2	3300.8	3087.6	3063.6	117.3

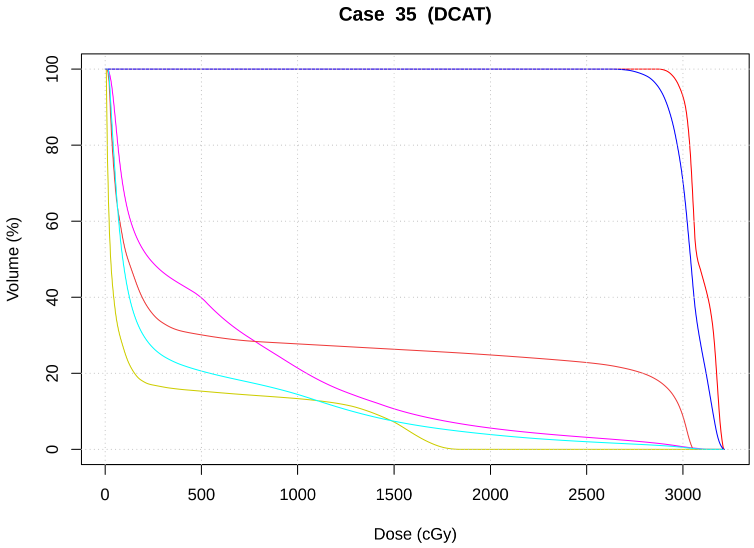

Structure	V [cm3]	Dmin	Dmax	Dmedian	Dmean	Dstd
CTV	29.3	2865.7	3215.4	3075.5	3095.6	72.0
lung	3053.9	11.6	3179.9	232.0	576.9	690.7
esophagus	35.9	11.3	3055.9	118.2	843.9	1205.9
spinal cord	73.5	5.7	1844.7	29.0	251.7	502.2
heart	749.8	13.4	3148.7	91.8	372.5	617.7
PTV	145.2	2614.0	3215.4	3039.7	3036.9	90.2
